# Charge Assisted S/Se Chalcogen Bonds in SAM Riboswitches: A Combined PDB and *ab Initio* Study

**DOI:** 10.1021/acschembio.1c00417

**Published:** 2021-08-24

**Authors:** María de las Nieves Piña, Antonio Frontera, Antonio Bauza

**Affiliations:** † Department of Chemistry, Universitat de les Illes Balears, Crta. de Valldemossa km 7.5, 07122 Palma (Baleares), Spain

## Abstract

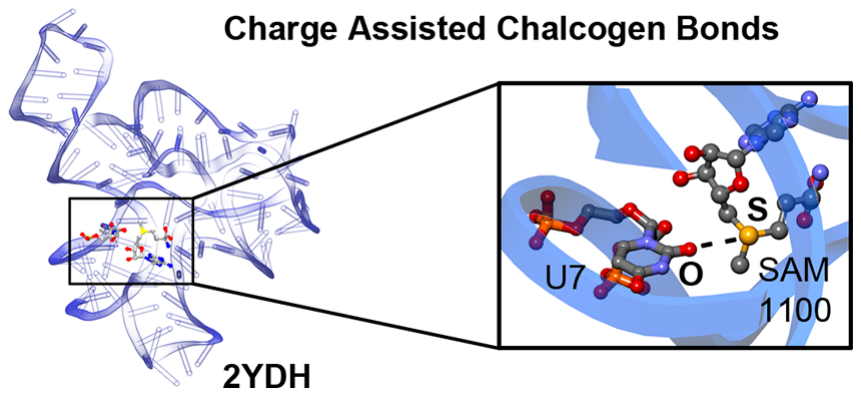

In this study, we provide experimental (Protein Data Bank (PDB) inspection) and theoretical (RI-MP2/def2-TZVP level of theory) evidence of the involvement of charge assisted chalcogen bonding (ChB) interactions in the recognition and folding mechanisms of S-adenosylmethionine (SAM) riboswitches. Concretely, an initial PDB search revealed several examples where ChBs between S-adenosyl methionine (SAM)/adenosyl selenomethionine (EEM) molecules and uracil (U) bases belonging to RNA take place. While these interactions are usually described as a merely Coulombic attraction between the positively charged S/Se group and RNA, theoretical calculations indicated that the σ holes of S and Se are involved. Moreover, computational models shed light on the strength and directionality properties of the interaction, which was also further characterized from a charge-density perspective using Bader’s “Atoms in Molecules” (AIM) theory, Non-Covalent Interaction plot (NCIplot) visual index, and Natural Bonding Orbital (NBO) analyses. As far as our knowledge extends, this is the first time that ChBs in SAM–RNA complexes have been systematically analyzed, and we believe the results might be useful for scientists working in the field of RNA engineering and chemical biology as well as to increase the visibility of the interaction among the biological community.

## Introduction

During the past decade, noncovalent interactions (NCIs) have started a fast growing revolution, which has led them to become essential resources of the chemist toolbox owing to their crucial role in several fields of modern chemistry, such as supramolecular chemistry,^1^ molecular recognition,^2^ and materials science.^3^ Despite the great importance that hydrogen bonding interactions (HB) play in many chemical and biological systems,^4,5^ such as in enzymatic chemistry and protein folding and binding phenomena,^6^ other noncovalent interactions based on the p-block of elements (aerogen,^7^ halogen,^8^ chalcogen,^9^ pnictogen,^10^ and tetrel bonds)^11^ have emerged as novel and powerful resources for rational drug design,^12−14^ molecular aggregation^15−17^ or even tuning self-assembly processes.^18−20^ Among them, chalcogen bonds (ChBs) have been studied both theoretically^21−24^ and experimentally in several areas of research, such as host–guest chemistry,^25,26^ crystal engineering and materials science,^27−29^ and catalysis.^30,31^ In biology, ChBs have been mainly studied in protein–ligand complexes,^32^ involving glucosidases,^33^ Zn finger proteins,^34^ C-Jun N-terminal kinase 3,^35^ iodothyronine deiodinase,^36^ and lysine methyltransferase SET7/9^37^ systems. However, their study and applications in the context of nucleic acid chemistry are scarce in the literature.

In this regard, S-adenosyl methionine (SAM) riboswitches are structured regulatory RNA elements controlling gene expression phenomena by directly reacting to variations in cellular conditions without the implication of proteins.^38^ More precisely, they usually refer to an RNA sequence bound to specific ligands (e.g., small metabolites or metal ions). RNA riboswitches’ main biological mission is intimately related to gene regulation and expression processes, including the control of mRNA degradation or alternative splicing.^39,40^ Their architecture is usually composed of two domains: (i) an upstream aptamer domain involved in ligand recognition and (ii) a downstream expression system. The latter can flip between “on” or “off” conformations, which affects the mechanism undertaken to carry out either transcription or translation. Moreover, binding of specific ligands^41−43^ (e.g., SAM) influences cross-talking between the two domains, leading to the activation of the expression platform system.

**Figure 1:**
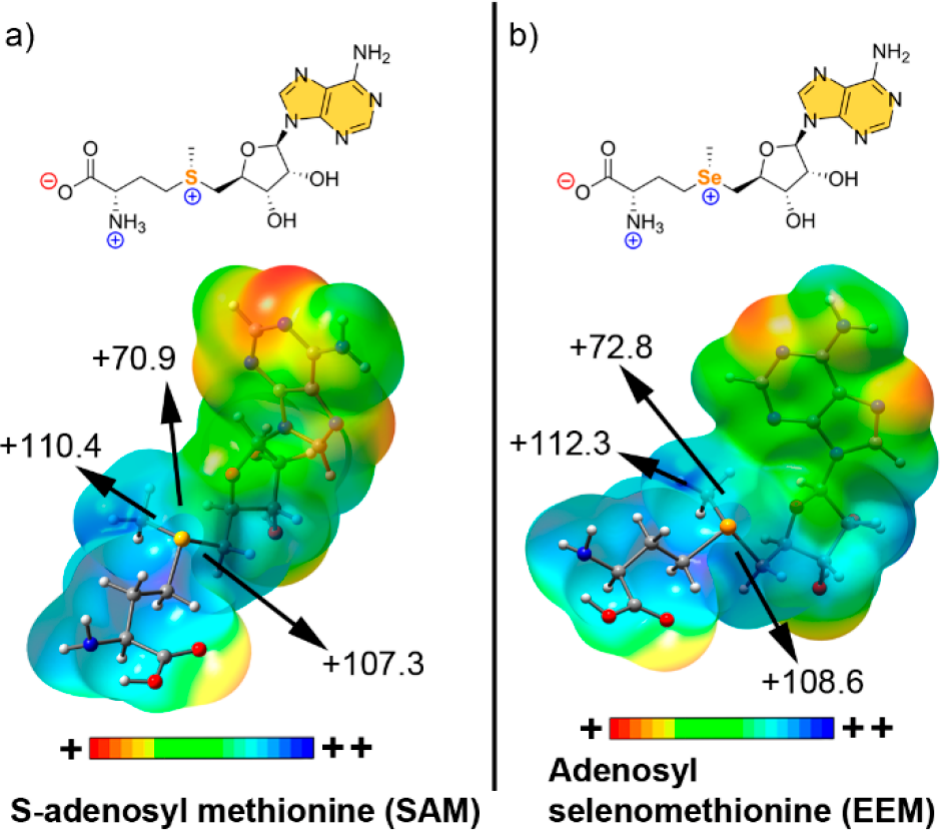
MEP surfaces of SAM (a) and EEM (b). Energies at selected points of the surface (0.001 au) are given in kcal·mol^–1^.

SAM (see Figure 1a) is a key metabolite in all living organisms, synthesized from methionine and ATP by SAM synthetase, and represents a universal methylation resource inside cells. To date, three distinct families of SAM riboswitches have been characterized: the SAM-I superfamily, encompassing the SAM-I (S-box), SAM-IV, and SAM-I/IV systems; the SAM-II superfamily, involving SAM-II and SAM-V families; and the SAM-III (or SMK-box) family.^44^ Usually, the main noncovalent driving force in SAM-RNA recognition is merely referred to as a favorable electrostatic interaction between the positively charged sulfonium group and an O atom from an uracil (U) base of RNA. However, a Molecular Electrostatic Potential (MEP) surface analysis of SAM and adenosyl selenomethionine (EEM) molecules (Figure 1) revealed that the most positive electrostatic potential regions of the sulphonium and selenium groups are located on the extension of the C–S and C–Se bonds, respectively, which are prototypical descriptors of σ holes. Therefore, the term charge assisted ChB should be used to describe this type of noncovalent binding, as it has been previously applied for protein–ligand complexes involving positively charged sulfur moieties.^33,37^

In this work, we have performed a combined crystallographic (Protein Data Bank (PDB) survey) and computational (RI-MP2/def2-TZVP level of theory) study to analyze for the first time the charge assisted ChBs established during SAM-RNA complex formation. To achieve this goal, we have inspected the PDB and found 54 X-ray crystal structures involving nucleic acids and SAM/EEM molecules, 28 of them exhibiting ChBs. Theoretical models based on these structures were created, and the strength and directionality of ChB interactions were theoretically evaluated. Additionally, the noncovalent interactions studied herein were further characterized by means of Bader’s “Atoms in Molecules” (AIM) theory, Non-Covalent Interaction plot (NCIplot) visual index, and Natural Bonding Orbital (NBO) analysis.

## Results and Discussion

The following examples were selected from the PDB survey to show a representative set of structures, since each of them belongs to a different SAM-riboswitch family (see Supporting Information for details regarding the creation of the theoretical models and Table S1 for energetic results regarding the rest of structures).

### Computations on Selected Examples from PDB

Structures 2YDH and 2YGH(45) correspond to the *T. tengcongensis* SAM-I riboswitch. The study from Schroeder and collaborators^45^ is focused on the analysis of an RNA kink turn (k-turn), which is a well-known structural motif that contributes to long-range interactions of RNA by the incorporation of a severe kink into its architecture. This type of RNA structural motif is involved in most aspects of RNA functionality, including translation, guided methylation and pseudouridylation mechanisms, spliceosome assembly, and genetic control.^46^

**Figure 2:**
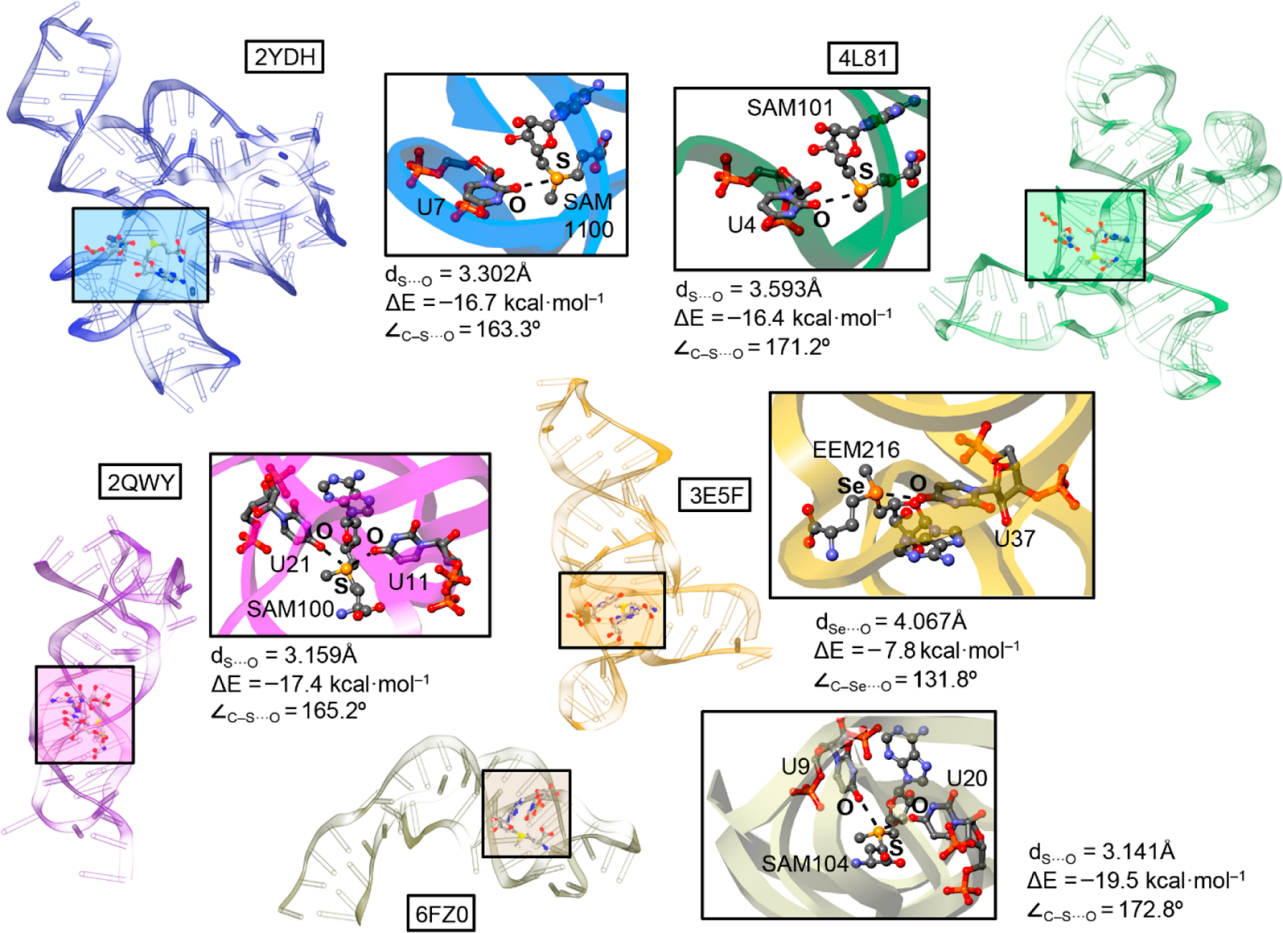
Examples of crystal structures exhibiting ChBs. The ChB interaction is magnified inside the square parts of the figure. The S···O and Se···O distances (d_S···O_ and d_Se···O_), the interaction energy values (ΔE) and C−S···O and C−Se···O angles (∠_C−S···O_ and ∠_C−Se···O_) are also indicated.

In their study, the authors used the SAM-I riboswitch as a model to analyze the influence of RNA tertiary interactions on the stabilization of a k-turn using a nonconsensus RNA sequence. They used isothermal titration calorimetry to measure the binding affinity of the SAM for the riboswitch, obtaining an experimental Δ*G*° value of −36 ± 1.2 kJ mol^–1^. As noticed in Figure 2a, the SAM molecule is interacting with the RNA in a way that an O atom from the OC carbonyl group of U7 is in close contact with the SAM’s sulphonium group (*d*_S···O_ = 3.302 Å). In addition, the C–S···O angle is close to linearity (163.3°), thus indicating that the O atom from U is maximizing the interaction with the S σ hole, in agreement with the MEP analysis shown above. The computed interaction energy for this charge assisted ChB resulted to be −16.7 kcal/mol, which is a moderately strong value. Interestingly, mutations on the k-turn sequence resulted in a complete absence of SAM binding; therefore, the formation of this structural RNA motif resulted in being key to the proper functioning of the RNA riboswitch and thus, to establishing charge assisted ChB.

The second example (structures 4L81 and 4OQU)^47^ involves the X-ray structure of an env87 (ΔU92) aptamer from the SAM-I/IV riboswitch. Concretely, Trausch and co-workers^47^ studied the structure of a member of the SAM-I/IV family containing an additional PK-2 subdomain to reveal its influence in facilitating both effector recognition and the regulatory switch. Despite the core architecture of the SAM-I/IV aptamer domain being similar to that of the SAM-I aptamers that have been previously determined,^48^ the crystal structure of env87 (ΔU92) revealed a completely novel peripheral architecture surrounding the core structure of the riboswitch.

To evaluate the influence of SAM binding on the structure of the env87 SAM-I/IV aptamer domain, the authors carried out selective 2-hydroxyl acylation analyzed by primer extension (SHAPE) chemical probing experiments in the absence and presence of SAM. The authors concluded that nucleotides G8 and A25 were directly involved in SAM recognition, thus showing the strongest degrees of protection. In addition, they observed that binding of SAM strongly promoted long-range interactions within the PK-2 subdomain that further act as a stabilization source. In this regard, a fact that passed unnoticed to the original authors is that the OC group from U4 is establishing a charge assisted ChB with the positive sulfur moiety of SAM (see Figure 2b). In this case, the C–S···O angle is very close to linearity (171.2°), and the calculated interaction energy resulted to be −16.4 kcal·mol^–1^. Thus, ChBs are involved in the stabilization of the PK-2 subdomain. This is important since PK-2 subdomain stabilization is required for this riboswitch to exert its regulatory activity.

The third structure (2QWY)^49^ encompasses the X-ray structure determination of a SAM-II riboswitch complexed to SAM found in the 5′ UTR of the *metX* gene present on an environmental sequence in the Sargasso Sea metagenome.^50^ In the study from Gilbert and collaborators,^49^ the SAM recognition was attributed to a plethora of SAM-RNA HBs involving the A base and the methione moiety of SAM. Interestingly, chemical probing using *N*-methylisatoic acid (NMIA) revealed a clear ligand-dependent stabilization of the RNA architecture. More precisely, differences were found in chemical probing of the unliganded and liganded structures, thus indicating that binding of SAM to the riboswitch clearly alters the local conformation of several adenosines (concretely, A19, A41, and A49).

The recognition of the sulphonium group was mentioned as an electrostatic interaction involving U11 and U21 bases. However, a close look reveals that the sulfonium group of SAM interacts with two different O atoms belonging to the OC carbonyl groups from U11 and U21 bases (Figure 2c), which are in close contact with the two accessible SAM σ holes, thus establishing two simultaneous charge assisted ChBs. In Figure 2c, only the data corresponding to the shortest ChB is shown (involving U21), exhibiting an interaction energy value of −17.4 kcal·mol^–1^ and a C–S···O angle of 165.2°.

The fourth selected example (structures 3E5C and 3E5F)^51^ belongs to the X-ray crystal structure of the SAM-III/SMK riboswitch. During their structural analysis, Lu and collaborators^51^ used Se to identify the position of the S atom in the SAM molecule (EEM in Figures 1 and 2d). In this regard, the positively charged selenium group of EEM establishes a charge assisted ChB with the OC group from U37, a fact that passed unnoticed to the original authors, which just merely attributed its recognition to favorable electrostatic interactions between both counterparts. In this case, the O atom is not pointing directly to a Se σ hole (C–Se···O angle of 131.8 degrees); instead it is placed above the Se atom, which also exhibits a positive MEP value, although of less magnitude than that of the Se–C σ holes (see Figure 1b), leading to a less favorable interaction energy value (−7.8 kcal·mol^–1^).

The last structure (6FZ0)^52^ corresponds to the SAM-V riboswitch. Concretely, the study from Huang and co-workers^52^ structurally characterizes this less known and abundant type of RNA assembly. The authors stated that the sulfonium group of SAM is mainly stabilized by favorable Coulombic interactions involving the S atom and the U9 and U20 bases. Similarly to 2QWY, two O atoms from two different OC carbonyl groups (U9 and U20) establish two simultaneous charge assisted ChBs with the two S σ holes of SAM. In Figure 2e, only the data corresponding to the ChB involving U9 is shown, resulting in an interaction strength of −19.5 kcal·mol^–1^.

### AIM and NCIplot Analyses

**Figure 3:**
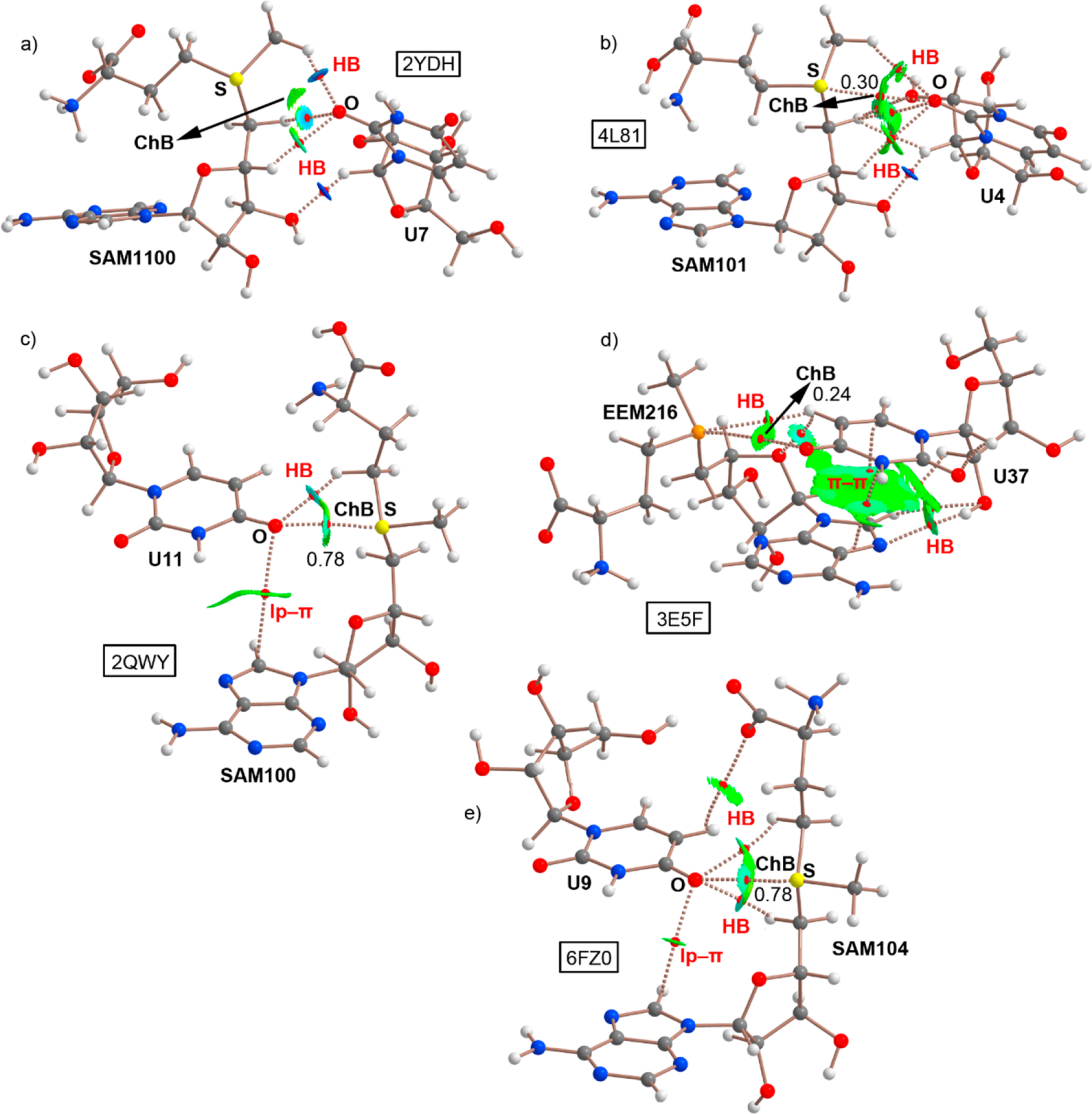
NCIplot analysis and AIM distribution of intermolecular bond critical points (BCP in red spheres) and bond paths in (a) 2YDH, (b) 4L81, (c) 2QWY, (d) 3E5F, and (e) 6FZ0 model structures. The value of density at the BCPs characterizing the ChB interaction is also indicated. Ancillary interactions are highlighted in red. NCIplot surfaces involving only intermolecular contacts between SAM/EEM and U bases are also indicated. NCIPlot color range −0.02 au ≤ (signλ2)ρ ≤ + 0.02 au. Isosurface value |RGD| = 0.5, and ρ cutoff = 0.04 au.

The QTAIM analysis^53^ of the ChBs gathered in structures 2YDH, 4L81, 2QWY, 3E5F, and 6FZ0 is shown in Figure 3. A close look reveals some interesting aspects to discuss. First, in all cases with the exception of 2YDH, a bond critical point (BCP) and a bond path connects the S/Se atom from SAM/EEM ligands to the OC carbonyl group of a U base, thus characterizing the ChB interaction. In the case of 2YDH, although no BCP was found, a greenish NCIplot isosurface can be observed between the S atom from SAM1100 and an O atom of U7, which confirms the presence and favorable nature of the ChB. In addition, in all complexes, ancillary interactions are undertaken. More precisely, in 2YDH a bifurcated hydrogen bond (HB) is established involving two CH groups from SAM1100 and an O atom from U7. Also, another HB is undergone involving the OH group from the pentose moiety of SAM and a CH group from the pentose ring of U7.

In the case of 4L81, an HB network is established between SAM101 and U4 moieties, involving O atoms from the pentose ring of SAM and from U7 as electron donors and the aliphatic CH groups from both molecules as acceptors. On the other hand, in the 2QWY structure, an ancillary HB is characterized by a BCP and a bond path connecting an O atom from U11 to a CH group from SAM100. In addition, an ancillary lone pair−π (lp−π) interaction is characterized by the presence of a BCP and a bond path connecting an O atom from U11 and a C atom from the adenine ring of SAM.

In the case of 3E5F, several ancillary HBs are established between the adenine and the pentose rings of EEM216 and U37. In addition, a π–π stacking interaction is also present involving the π systems of A and U, as denoted by the two BCPs and bond paths connecting the N and C atoms from both moieties. Last, in the 6FZ0 structure, three ancillary HBs are characterized by the presence of three BCPs and bond paths connecting SAM104 CH and carboxylate groups with (i) an O atom and (ii) a CH group from U9. Furthermore, an lp−π interaction is also undertaken involving an O atom from U9 and a C atom from the adenine ring of SAM. It is also worthy to emphasize that both 6FZ0 and 2QWY structures exhibited the largest ρ values at the BCP that characterizes the ChB, indicating that the ChBs present in these two complexes are stronger than those present in 2YDH, 4L81, and 3E5F structures. Finally, the values of the Laplacian are in all cases positive, as it is known for closed shell calculations.

In order to provide quantitative evidence of the impact of ChBs on the stabilization of the noncovalent complexes studied herein, we have also evaluated the individual contribution of each noncovalent force (ChB, HB, lp−π, and π–π stacking) in 2YDH, 4L81, 2QWY, 3E5F, and 6FZ0 structures, and the results are gathered in Table 1. In the case of the HB interactions, the energetic contribution was estimated using the formula developed by Espinoza and collaborators.^54^ On the other hand, the ChB, lp−π, and π–π contributions were estimated using additional theoretical models (see SI for more details).

**Table 1:** Energetic Contribution (in kcal·mol^–1^) of Each Noncovalent Force: Chalcogen Bonding (ChB), Hydrogen Bonding (HB), Lone Pair−π (lp−π), and π–π Stacking in 2YDH, 4L81, 2QWY, 3E5F, and 6FZ0 Complexes

PDB ID	ChB	HB	lp−π	π–π stacking
2YDH	–5.5	–11.2		
4L81	–10.0	–6.4		
2QWY	–13.5	–1.8	–2.0	
3E5F^a^	–7.3	–2.3		–6.5
6FZ0	–14.3	–2.8	–2.5	

a In the 3E5F structure, the sum of ChB, HB, and π–π contributions exceeds the value given in Figure 2, where a specific model was used to compute the interaction energy (see the Methods).

As noticed in Table 1, in all cases except for the 2YDH structure, the ChB is the most prominent interaction governing the stability of the complex (e.g., −10.0 kcal/mol in 4L81 or −14.3 kcal/mol in 6FZ0). In the 2YDH structure, the contribution to the total interaction energy of the ChB is around 33%. In addition, in 2QWY and 6FZ0 the lp−π interactions are of similar strength (−2.0 and −2.5 kcal/mol, respectively) to that of the HBs present in these structures (−1.8 and −2.8 kcal/mol, respectively). These results highlight the impact of the ChBs in directing the formation of the SAM–RNA complexes studied herein, being a noticeable director force guiding the molecular recognition phenomena.

The NCIplot analyses are a useful identification tool which allows intuitive establishment of the location of noncovalent interactions in real space as well an unveiling of their favorable/unfavorable nature. The NCIplot selected examples confirm the attractive nature of the charge assisted ChBs studied herein (as indicated by the greenish isosurfaces located between the S/Se atoms and the O atoms from U). Finally, the favorable nature of ancillary HB, lp−π, and π–π interactions is confirmed by presence of bluish and greenish isosurfaces placed between both SAM/Se-SAM and U counterparts.

### NBO Analysis

To further investigate the participation of orbital contributions in the stabilization of the noncovalent complexes studied, we carried out NBO calculations^55^ focusing on a second order perturbation analysis that is useful in evaluating donor–acceptor interactions (see Table 2 and Table S2 in the Supporting Information).

**Table 2:** Donor and Acceptor NBOs with Indication of the Second-Order Interaction Energy *E*^(2)^ and Donor and Acceptor Orbitals for the Selected PDB Structures^a^

2YDH	LP O	BD* S–C	0.22
4L81	LP O	BD* S–C	0.12
2QWY	LP O	BD* S–C	0.80
6FZ0	LP O	BD* S–C	0.98

a LP and BD* stand for lone pair and antibonding orbital, respectively. Energy values are in kcal/mol. In the 3E5F complex, no orbital contribution involving a BD* Se–C orbital above the energetic threshold (0.05 kcal·mol^–1^) was found.

**Figure 4:**
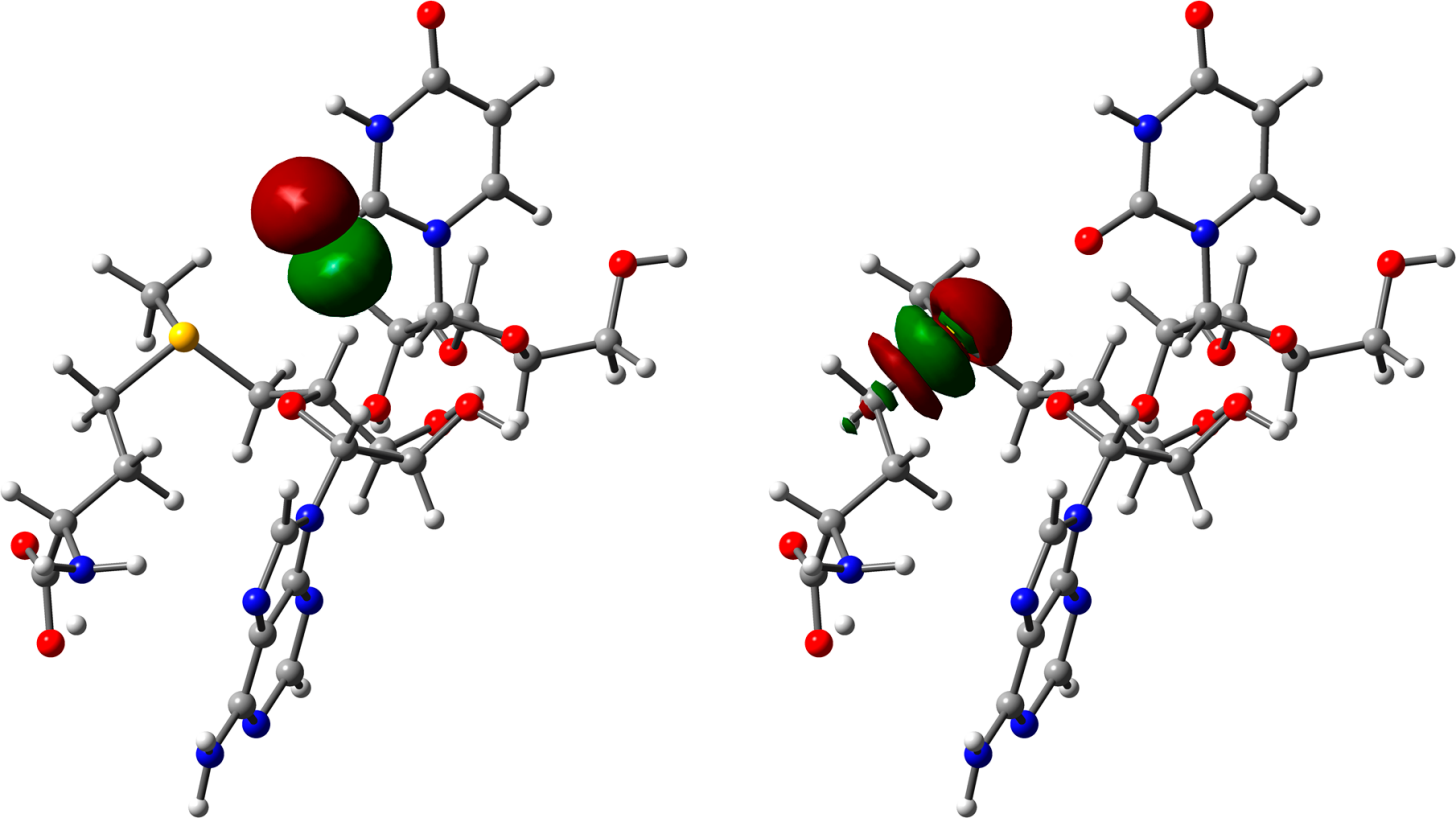
NBO plots of the donor (LP O, left) and acceptor (BD* S–C, right) orbitals involved in the ChB interaction of 2YDH structure.

The results reveal an orbital contribution from the lone pair (LP) of the O atom belonging to the carbonyl moiety of U to an antibonding (BD*) S–C orbital, ranging from 0.1 to 1 kcal·mol^–1^. Although their impact to the total interaction energy is low (<10%), this analysis serves as further confirmation of the σ-hole nature of the charge assisted ChBs studied herein. Additionally, it marks the importance of orbital contributions to the stabilization of the complexes despite the strong role that electrostatics play in SAM–RNA binding. This aspect can also be confirmed by the high directionality exhibited by the ChBs present herein (showing C–S···O angles comprised between 160 and 170° in Figure 1); thus, the ChBs might act as a subtle director of the SAM–RNA recognition phenomena. Finally, using the structure 2YDH as a representative case, we have also included a graphical representation of the donor (LP O) and acceptor (BD* S–C) orbitals involved in the formation of the ChBs studied herein (see Figure 4).

## Conclusions

In conclusion, we have estimated the stability of a series of charge assisted chalcogen bonds in SAM riboswitches. A general PDB inspection revealed 28 X-ray structures exhibiting ChBs between S/Se atoms from SAM/EEM molecules and a U base from RNA. Theoretical models indicate that the interaction is of a moderately strong nature (owing to the positively charge of SAM/EEM moiety) and was characterized using AIM and NCIplot methodologies. Finally, NBO calculations show a minor role for orbital contributions to the global stabilization of the complexes studied herein. We hope the findings gathered in this work will be useful for the community working on RNA engineering and chemical biology as well as to make more visible the ChB interaction among the biological community.

## Methods

### PDB Analysis

#### Creation of the Theoretical PDB Models

The Protein Data Bank was interrogated (May 2020) by manually inspecting all of the X-ray crystal structures containing a SAM–RNA complex. The criteria used to classify an interaction as a ChB follows:

1. *d*_S···A_ ≤ sum of vdW radii +0.5
2. ∠_C–S···A_ between 150 and 180°

**Figure 5:**
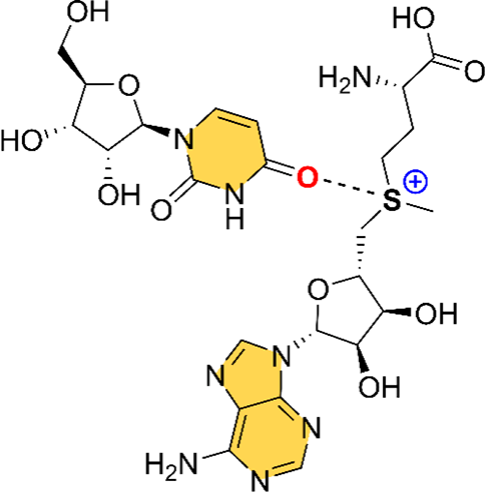
Schematic representation of the theoretical models used to compute the charge assisted ChB energies.

Structure 3E5F (involving Se) was considered for this study despite its poor directionality (131.8°). A general view of the charge assisted ChB interaction studied herein is shown in Figure 5.

All computational models except for the 6YLB structure were built using the Figure 4 scheme as a template. The charge of the complex was set to +1 in all cases, and the monomers were, on one hand, the SAM/EEM molecules and, on the other hand, the U base containing the sugar moiety. The phosphate groups connected to the pentose were replaced by OH groups to get rid of the strong electrostatics that dominate the interaction.

**Figure 6:**
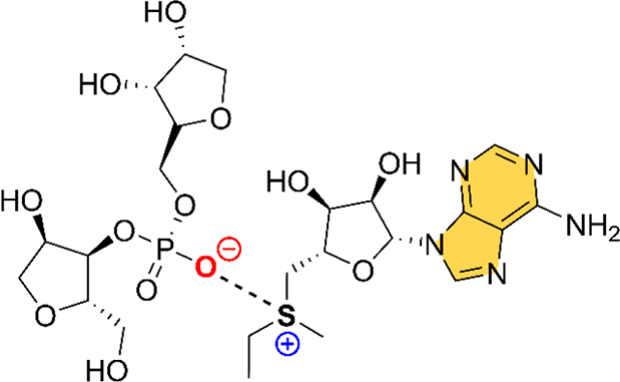
Schematic representation of the theoretical model used in the 6YLB structure.

In the 6YLB structure, the model gathered in Figure 6 was used. In this particular case, the interaction involves an O atom of the phosphate backbone as an electron donor. Thus, the negatively charged nature of the phosphate was preserved, leading to a neutral charged complex.

Finally, some modifications from the general scheme were made to 3E5F and 6YLB structures to evaluate the ChB interaction. In both cases, the adenine portion of EEM and SAM molecules was replaced by a H atom to (i) avoid evaluating a π–π stacking interaction with the π system of U in 3E5F and (ii) avoid evaluating a lone pair−π interaction with the O atom from the pentose moiety of the electron donor partner. The interaction energy values given in Figure 1 and Table S1 correspond to the modified models. The Cartesian coordinates of both models (complete and modified) are also gathered in the SI. Finally, to estimate the contribution of each noncovalent interaction in structures 2QWY and 6FZ0, an additional theoretical model was created (see SI for Cartesian coordinates) where the adenine ring of the SAM moiety was replaced by a H atom.

### Computations

The energies of all complexes included in this study were computed at the RI-MP2^56^/def2-TZVP^57^ level of theory. The calculations were performed by using the program TURBOMOLE, version 7.0.^58^ Initially, the H atoms from the X-ray crystal structure models (see SI for Cartesian coordinates) were optimized at the BP86^59^-D3^60^/def2-SVP^57^ level of theory. These geometries were taken as a starting point for single point calculations at the RI-MP2/def2-TZVP level of theory. The MEP (Molecular Electrostatic Potential) surfaces were computed at the RI-MP2/def2-TZVP level of theory by means of the Gaussian 16 calculation package.^61^ Bader’s “Atoms in Molecules” theory has been used to study the interactions discussed herein by means of the AIMall calculation package.^62^ The NBO analyses was performed at the HF/def2-TZVP level of theory. The calculations for the wave function analysis have been carried out at the B3LYP/def2-TZVP level of theory using Gaussian 16 software. TheNCIplot^63^ isosurfaces correspond to both favorable and unfavorable interactions, as differentiated by the sign of the second density Hessian eigenvalue and defined by the isosurface color. The color scheme is a red–yellow–green–blue scale with red for repulsive (ρ_cut_^+^) and blue for attractive (ρ_cut_^–^) NCI interaction density. Yellow and green surfaces correspond to weak repulsive and weak attractive interactions, respectively.

#### Results from the PDB Survey

##### SAM–RNA ChB Containing Structures

2YDH, 4L81, 2QWY, 3E5F (Se), 6FZ0, 2YGH, 3GX5, 3GX6, 3IQN, 3IQR, 7JYY, 3V7E, 4AOB, 4KQY, 4OQU, 6YLB, 3E5C, 5FJC, 5FK1, 5FK2, 5FK3, 5FK4, 5FK5, 5FK6, 5FKD, 5FKE, 5FKG, 5FKH.

## Supporting Information Available

Tables S1 and S2 and Cartesian coordinates of the PDB models used (PDF)
