# Supplementary material for: Charge Assisted S/Se Chalcogen Bonds in SAM Riboswitches: A Combined PDB and ab Initio Study
Source: ACS Chem Biol. 2021 Aug 24;16(9):1701–8. doi: 10.1021/acschembio.1c00417 (PMC8525861; doi:10.1021/acschembio.1c00417)
Supplement: Supplementary file 1 — cb1c00417_si_001.pdf [file cb1c00417_si_001.pdf]

Charged assisted S/Se Chalcogen Bonds in SAM riboswitches: A combined PDB and *ab initio* study.

María de las Nieves Piña,[a] Antonio Frontera[a] and Antonio Bauza[a,*]

[a]*Department of Chemistry. Universitat de les Illes Balears*
Crta. de Valldemossa km 7.5. 07122 Palma de Mallorca (Spain)
E-mail: antonio.bauza@uib.es

Table S1. Interaction energies (ΔE_{BSSE} corrected in kcal·mol^{-1}). electron rich atom (E.l.r.). equilibrium distances (in Å) and values of the density at the ChB bond critical point ($\rho \cdot 10^2$ in a.u.) at the RI–MP2/def2–TZVP level of theory.

PDBID	E.l.r.	ΔE_{BSSE}	R	$\rho \cdot 10^2$
**3GX5**	O(U7)	–14.2	3.509	-[a]
**3GX6**	O(U7)	–15.8	3.009	1.01
**3IQN**	O(U7)	–17.3	3.296	-[a]
**3IQR**	O(U7)	–9.7	3.398	-[a]
**7JYY**	O(U2)	–12.0	4.046	0.18
**3V7E**	O(U320)	–15.4	3.636	0.38
**4AOB**	O(U7)	–18.5	3.222	-[a]
**4KQY**	O(U7)	–12.8	3.645	-[a]
**4OQU**	O(U4)	–19.2	3.686	-[a]
**6YLB**	OP(G25)	–62.9	3.440	0.60
**2YGH**	O(U7)	–19.0	3.495	-[a]
**3E5C**	O(U37)	–15.3	4.108	-[a]
**5FJC**	O(U7)	–16.5	3.739	-[a]
**5FK1**	O(U7)	–16.2	3.503	0.36
**5FK2**	O(U7)	–18.1	3.827	-[a]
**5FK3**	O(U7)	–17.3	3.522	-[a]
**5FK4**	O(U7)	–16.3	3.646	-[a]
**5FK5**	O(U7)	–16.8	3.775	-[a]
**5FK6**	O(U7)	–15.7	4.085	-[a]
**5FKD**	O(U7)	–9.6	4.136	-[a]
**5FKE**	O(U7)	–13.7	4.084	-[a]
**5FKG**	O(U7)	–8.4	3.816	-[a]
**5FKH**	O(U7)	–17.2	3.976	-[a]

[a]In these structures no CP connecting the S atom of SAM and the O atom from U was found.

Table S2. Donor and acceptor NBOs with indication of the second-order interaction energy $E^{(2)}$ and donor and acceptor orbitals for additional PDB structures. Energy values are in kcal/mol.

PDBID	Donor	Acceptor	$E^{(2)}$
**3GX5**	LP O	BD*S–C	0.16
**3GX6**	LP O	BD*S–C	0.95
**3IQN**	LP O	BD*S–C	0.29
**3IQR**	LP O	BD*S–C	0.21
**7JYY**	LP O	BD*S–C	0.06
**3V7E**	BD C–O	BD*S–C	0.07
**4AOB**	LP O	BD*S–C	0.56
**4KQY**[a]	-	-	-
**4OQU**	LP O	BD*S–C	0.06
**6YLB**	LP O	BD*S–C	0.67
**2YGH**	LP O	BD*S–C	0.13
**3E5C**[a]	-	-	-
**5FJC**	LP O	BD*S–C	0.06
**5FK1**	LP O	BD*S–C	0.18
**5FK2**[a]	-	-	-
**5FK3**	LP O	BD*S–C	0.17
**5FK4**	LP O	BD*S–C	0.07
**5FK5**[a]	-	-	-
**5FK6**[a]	-	-	-
**5FKD**[a]	-	-	-
**5FKE**[a]	-	-	-
**5FKG**	LP O	BD*S–C	0.06
**5FKH**[a]	-	-	-

[a]In this complex no orbital contribution involving a BD* S–C orbital above threshold (0.05 kcal·mol^{-1}) was found.

Cartesian coordinates of PDB models

2QWY (complete)

O	-4.9767804	-1.3802996	-3.5627113
C	-6.1767804	-0.6172996	-3.5187113
C	-5.8807804	0.8047004	-3.1007113
O	-5.3947804	0.8237004	-1.7317113
C	-4.7827804	1.5237004	-3.8707113
O	-5.2627804	2.0077004	-5.1247113
C	-4.4327804	2.6497004	-2.9007113
O	-5.3537804	3.7237004	-2.8857113
C	-4.4987804	1.9137004	-1.5617113
N	-3.1787804	1.3937004	-1.1797113
C	-2.3047804	2.2747004	-0.5957113
O	-2.6077804	3.4257004	-0.3477113
N	-1.0657804	1.7617004	-0.3077113
C	-0.6327804	0.4787004	-0.5337113
O	0.5322196	0.1787004	-0.2827113
C	-1.6087804	-0.3812996	-1.1157113
C	-2.8207804	0.0957004	-1.4117113
N	0.7082196	-2.0242996	-3.8907113
C	2.1222196	-1.6252996	-3.7017113
C	2.8332196	-1.7302996	-4.9947113
O	4.0582196	-1.7962996	-5.0197113
O	2.1872196	-1.7822996	-6.0187113
C	2.8482196	-2.4982996	-2.6757113
C	2.0482196	-2.5542996	-1.3827113
S	2.9692196	-1.8132996	-0.0137113
C	4.5722196	-2.6602996	-0.1597113
C	2.2122196	-2.7352996	1.3672887
C	2.6832196	-2.1582996	2.7012887
O	2.4852196	-0.7612996	2.6602887
C	1.8262196	-2.6952996	3.8712887
O	2.7132196	-3.3152996	4.8122887
C	1.2342196	-1.4062996	4.5022887
O	0.9782196	-1.4592996	5.8972887
C	2.2412196	-0.3602996	3.9952887
N	1.7472196	1.0057004	3.9882887
C	0.4962196	1.4377004	3.6352887
N	0.4322196	2.7347004	3.7302887
C	1.6312196	3.2157004	4.1442887
C	2.1662196	4.5017004	4.4132887
N	1.3852196	5.6297004	4.2562887
N	3.4552196	4.6037004	4.8142887

C	4.2362196	3.5287004	4.9482887
N	3.7672196	2.3047004	4.7252887
C	2.4912196	2.1067004	4.3172887
H	-6.9188067	-1.0488080	-2.8059066
H	-6.6621834	-0.5529121	-4.5215015
H	-6.8193840	1.4004828	-3.1732019
H	-3.9151115	0.8567921	-4.0440018
H	-3.4023074	3.0226515	-3.0923404
H	-4.8786930	4.5140056	-2.5735616
H	-4.8324075	2.5911526	-0.7507988
H	-1.3566452	-1.4342090	-1.2917755
H	-3.6123127	-0.5189017	-1.8733546
H	0.0263416	-1.4744860	-3.3582352
H	0.5401080	-3.0255351	-3.7431401
H	2.1359396	-0.5632609	-3.3733167
H	3.8801720	-2.1129463	-2.5873751
H	2.9685935	-3.5201159	-3.0902601
H	1.7796886	-3.5855512	-1.0781174
H	1.1328155	-1.9277289	-1.3976254
H	5.0914134	-2.5380073	0.8105940
H	4.4337081	-3.7312543	-0.3998673
H	5.1610285	-2.1493002	-0.9420886
H	2.4341288	-3.8151529	1.2541549
H	1.1294170	-2.5351101	1.2352322
H	3.7457485	-2.4256590	2.9161430
H	1.0327493	-3.4020867	3.5352381
H	2.1561816	-3.6881276	5.5244007
H	0.2545345	-1.1965337	4.0267771
H	1.8453068	-1.5161079	6.3488910
H	3.1807958	-0.3582216	4.5994030
H	-0.3060567	0.7639936	3.3131955
H	1.7594406	6.5277422	4.5610591
H	5.2829941	3.6569088	5.2699674
H	-5.7171436	2.8515267	-4.9160087
H	-5.1978916	-2.2687797	-3.8924287
H	-0.4096383	2.4123830	0.1391444
H	0.3878261	5.5191416	4.0833011
H	1.2166370	-1.7922647	-5.7582068

2QWY (modified)

O	-4.97678040	-1.38029960	-3.56271130
C	-6.17678040	-0.61729960	-3.51871130
C	-5.88078040	0.80470040	-3.10071130
O	-5.39478040	0.82370040	-1.73171130

C	-4.78278040	1.52370040	-3.87071130
O	-5.26278040	2.00770040	-5.12471130
C	-4.43278040	2.64970040	-2.90071130
O	-5.35378040	3.72370040	-2.88571130
C	-4.49878040	1.91370040	-1.56171130
N	-3.17878040	1.39370040	-1.17971130
C	-2.30478040	2.27470040	-0.59571130
O	-2.60778040	3.42570040	-0.34771130
N	-1.06578040	1.76170040	-0.30771130
C	-0.63278040	0.47870040	-0.53371130
O	0.53221960	0.17870040	-0.28271130
C	-1.60878040	-0.38129960	-1.11571130
C	-2.82078040	0.09570040	-1.41171130
N	0.70821960	-2.02429960	-3.89071130
C	2.12221960	-1.62529960	-3.70171130
C	2.83321960	-1.73029960	-4.99471130
O	4.05821960	-1.79629960	-5.01971130
O	2.18721960	-1.78229960	-6.01871130
C	2.84821960	-2.49829960	-2.67571130
C	2.04821960	-2.55429960	-1.38271130
S	2.96921960	-1.81329960	-0.01371130
C	4.57221960	-2.66029960	-0.15971130
C	2.21221960	-2.73529960	1.36728870
C	2.68321960	-2.15829960	2.70128870
O	2.48521960	-0.76129960	2.66028870
C	1.82621960	-2.69529960	3.87128870
O	2.71321960	-3.31529960	4.81228870
C	1.23421960	-1.40629960	4.50228870
O	0.97821960	-1.45929960	5.89728870
C	2.24121960	-0.36029960	3.99528870
H	-6.91880670	-1.04880800	-2.80590660
H	-6.66218340	-0.55291210	-4.52150150
H	-6.81938400	1.40048280	-3.17320190
H	-3.91511150	0.85679210	-4.04400180
H	-3.40230740	3.02265150	-3.09234040
H	-4.87869300	4.51400560	-2.57356160
H	-4.83240750	2.59115260	-0.75079880
H	-1.35664520	-1.43420900	-1.29177550
H	-3.61231270	-0.51890170	-1.87335460
H	0.02634160	-1.47448600	-3.35823520
H	0.54010800	-3.02553510	-3.74314010
H	2.13593960	-0.56326090	-3.37331670
H	3.88017200	-2.11294630	-2.58737510
H	2.96859350	-3.52011590	-3.09026010

H	1.77968860	-3.58555120	-1.07811740
H	1.13281550	-1.92772890	-1.39762540
H	5.09141340	-2.53800730	0.81059400
H	4.43370810	-3.73125430	-0.39986730
H	5.16102850	-2.14930020	-0.94208860
H	2.43412880	-3.81515290	1.25415490
H	1.12941700	-2.53511010	1.23523220
H	3.74574850	-2.42565900	2.91614300
H	1.03274930	-3.40208670	3.53523810
H	2.15618160	-3.68812760	5.52440070
H	0.25453450	-1.19653370	4.02677710
H	1.84530680	-1.51610790	6.34889100
H	3.18079580	-0.35822160	4.59940300
H	-5.71714360	2.85152670	-4.91600870
H	-5.19789160	-2.26877970	-3.89242870
H	-0.40963830	2.41238300	0.13914440
H	1.21663700	-1.79226470	-5.75820680
H	1.87733366	0.64591133	3.99013242

2YDH

O	2.7480242	-6.0359698	-3.5449728
C	3.4630242	-4.8429698	-3.3559728
C	3.0020242	-4.0319698	-2.1609728
O	1.7420242	-3.3489698	-2.4169728
C	2.7630242	-4.7929698	-0.8669728
O	4.0030242	-5.0969698	-0.2389728
C	1.9680242	-3.7249698	-0.1329728
O	2.7790242	-2.6479698	0.2900272
C	1.0110242	-3.2509698	-1.2129728
N	-0.2209758	-4.0839698	-1.2909728
C	-1.3239758	-3.7809698	-0.4969728
O	-1.3929758	-2.8439698	0.2890272
N	-2.3759758	-4.6399698	-0.6549728
C	-2.4449758	-5.7289698	-1.4779728
O	-3.4589758	-6.3789698	-1.4939728
C	-1.2929758	-5.9959698	-2.2629728
C	-0.2489758	-5.1759698	-2.1409728
N	-1.4249758	5.0280302	2.8970272
C	-1.2269758	3.7300302	3.6130272
C	0.1460242	3.6890302	4.3200272
O	0.4730242	2.7280302	5.0230272
O	0.9740242	4.6130302	4.2200272
C	-1.3999758	2.5770302	2.6300272

C	-1.7059758	1.2370302	3.2780272
S	-1.8899758	-0.1149698	2.0800272
C	-1.3979758	-1.5359698	3.0970272
C	-0.5099758	0.0260302	0.9330272
C	-1.0379758	0.4410302	-0.4269728
O	-1.9129758	1.5560302	-0.3539728
C	0.0840242	0.8190302	-1.3629728
O	0.6120242	-0.3099698	-1.9979728
C	-0.6299758	1.7210302	-2.3229728
O	-1.1979758	0.9410302	-3.3579728
C	-1.7229758	2.3730302	-1.4859728
N	-1.3559758	3.7380302	-1.0639728
C	-2.2019758	4.8190302	-1.0759728
N	-1.5439758	5.9110302	-0.6279728
C	-0.2919758	5.5500302	-0.3259728
C	0.7690242	6.2870302	0.1670272
N	0.6340242	7.5790302	0.4260272
N	1.9740242	5.6500302	0.3720272
C	2.0990242	4.2970302	0.0920272
N	1.0180242	3.5640302	-0.3829728
C	-0.1579758	4.1840302	-0.5899728
H	4.5474096	-5.0453698	-3.1711882
H	3.4091752	-4.1700989	-4.2457166
H	3.7849480	-3.2649176	-1.9687500
H	2.1582518	-5.7050105	-1.0578820
H	1.3942978	-4.1513912	0.7295636
H	3.6359851	-3.0557864	0.5450320
H	0.6927233	-2.2023031	-1.0590045
H	-1.3019498	-6.8516713	-2.9462651
H	0.6616350	-5.3060620	-2.7455148
H	-0.4381234	5.4611573	3.1603794
H	-2.1797549	5.6238117	3.2697606
H	-2.0020655	3.6746204	4.4058519
H	-0.4726909	2.5116030	2.0205439
H	-2.2101259	2.8127664	1.9045503
H	-0.8882300	1.0136217	4.0045875
H	-2.6725975	1.2497118	3.8232246
H	-0.3794932	-1.3718803	3.4972057
H	-2.1361394	-1.6286290	3.9156968
H	-1.4446334	-2.4044860	2.4117964
H	0.2428756	0.7273048	1.3448963
H	-0.0720387	-0.9876739	0.8471734
H	-1.5832882	-0.4319317	-0.8581619
H	0.8745141	1.3963844	-0.8423888

H	0.0482078	-0.4523175	-2.7926286
H	0.0598111	2.4884228	-2.7339238
H	-1.0611346	1.3837501	-4.2135071
H	-2.6635837	2.4622394	-2.0682800
H	-3.2429605	4.7709875	-1.4173903
H	1.4471853	8.1164133	0.7306264
H	2.9883234	-6.4123574	-4.4092922
H	3.8617994	-5.8374653	0.3783176
H	-1.4937507	4.9861393	1.8617585
H	-3.2110047	-4.4476962	-0.0931129
H	-0.2247881	8.0601260	0.1598644
H	3.0617719	3.7948323	0.2594173

2YGH

O	5.6489701	-2.9437010	3.6336558
C	4.5409701	-3.8087010	3.3606558
C	3.6869701	-3.1807010	2.2726558
O	3.3159701	-1.8047010	2.6066558
C	4.4239701	-3.0587010	0.9446558
O	4.4379701	-4.3077010	0.2786558
C	3.5869701	-1.9957010	0.2626558
O	2.3039701	-2.4877010	-0.0433442
C	3.3919701	-1.0257010	1.4206558
N	4.3559701	0.1412990	1.5436558
C	4.2109701	1.1952990	0.6586558
O	3.3709701	1.2052990	-0.2313442
N	5.0899701	2.2482990	0.8236558
C	6.0899701	2.3852990	1.7826558
O	6.8049701	3.4092990	1.8126558
C	6.1619701	1.2402990	2.6696558
C	5.3209701	0.1962990	2.5276558
N	-4.2250299	1.7482990	-2.8943442
C	-3.1970299	1.2542990	-3.8673442
C	-3.7050299	0.0452990	-4.6623442
O	-2.9680299	-0.5287010	-5.4693442
O	-4.8560299	-0.3827010	-4.5343442
C	-1.8290299	1.0062990	-3.2093442
C	-1.0850299	2.3112990	-2.8713442
S	0.4919701	2.1722990	-1.9623442
C	1.5319701	1.3522990	-3.1923442
C	0.1929701	0.7712990	-0.8493442
C	-0.5020299	1.2642990	0.4046558
O	-1.7590299	1.8572990	0.1196558
C	-0.8000299	0.1512990	1.3826558

O	0.3359701	-0.1277010	2.1736558
C	-1.9550299	0.7382990	2.1706558
O	-1.4490299	1.5012990	3.2426558
C	-2.6340299	1.7122990	1.2186558
N	-4.0220299	1.3392990	0.8386558
C	-5.0870299	2.1742990	0.9666558
N	-6.2200299	1.5432990	0.5636558
C	-5.9140299	0.3112990	0.1646558
C	-6.7110299	-0.7227010	-0.3453442
N	-8.0270299	-0.5677010	-0.5193442
N	-6.1120299	-1.9197010	-0.6793442
C	-4.7500299	-2.0607010	-0.5013442
N	-3.9680299	-1.0327010	0.0066558
C	-4.5340299	0.1592990	0.3316558
H	5.3504036	-0.6862636	3.1750312
H	4.9819633	3.0151507	0.1660293
H	6.9124907	1.2522249	3.4584633
H	2.4231667	-0.5275363	1.2788303
H	4.0727734	-1.5183467	-0.6066187
H	2.4333818	-3.3859115	-0.3834672
H	5.4423762	-2.6790280	1.1394605
H	2.7745982	-3.7821345	2.1296834
H	3.9309401	-3.9675784	4.2678521
H	4.8607059	-4.7951952	2.9777013
H	-3.8864857	2.5609056	-2.3880583
H	-4.4445346	1.0150226	-2.2216236
H	-3.0863861	2.0302479	-4.6460560
H	-1.2290393	0.3866895	-3.8968431
H	-2.0237093	0.4277896	-2.2940308
H	-1.6912139	2.9511504	-2.2127272
H	-0.8254768	2.8927054	-3.7708938
H	2.4839526	1.1452279	-2.6809505
H	1.6891762	2.0555827	-4.0205865
H	1.0481836	0.4304871	-3.5386681
H	1.2052710	0.4273289	-0.5767112
H	-0.3543190	-0.0147126	-1.3837045
H	0.1516623	1.9973471	0.9149564
H	-1.1635202	-0.7364014	0.8351927
H	0.4181268	-1.0776733	2.3189529
H	-2.6570288	-0.0430422	2.5140888
H	-0.6571784	1.0398578	3.5587692
H	-2.7383185	2.6734385	1.7456981
H	-5.0389453	3.1879784	1.3542124
H	-7.1444174	1.9659948	0.5747093

H	-8.3150733	0.3706970	-0.2302111
H	-4.2652398	-3.0056711	-0.7549689
H	5.2040732	-4.3462866	-0.3028380
H	6.2247948	-3.3780612	4.2707960
H	-4.9632050	-1.1357340	-5.1388881

3E5C

O	-5.4607797	-1.9558423	-2.2656594
C	-5.9557797	-1.0738423	-3.2766594
C	-4.8957797	-0.8658423	-4.3526594
O	-4.0147797	0.1931577	-3.9036594
C	-3.9627797	-2.0318423	-4.6876594
O	-3.6667797	-2.0278423	-6.1056594
C	-2.7087797	-1.6898423	-3.8716594
O	-1.5427797	-2.3188423	-4.3696594
C	-2.6717797	-0.1828423	-4.1096594
N	-1.8097797	0.6911577	-3.2196594
C	-0.8337797	1.4941577	-3.8056594
O	-0.6087797	1.5361577	-5.0096594
N	-0.1157797	2.2561577	-2.9186594
C	-0.2847797	2.3021577	-1.5366594
O	0.4342203	3.0361577	-0.8626594
C	-1.3177797	1.4571577	-1.0026594
C	-2.0287797	0.7051577	-1.8496594
N	5.0542203	1.9151577	3.9183406
C	3.7052203	2.3941577	4.3533406
C	3.5222203	2.3961577	5.8843406
O	4.4402203	2.0551577	6.6443406
O	2.4542203	2.7521577	6.4113406
C	2.6262203	1.5731577	3.6563406
C	1.4002203	2.4041577	3.2803406
S	-0.0997797	1.4611577	2.8943406
C	-1.1817797	1.9561577	4.2593406
C	0.3142203	-0.2228423	3.4463406
C	-0.4577797	-1.3088423	2.7053406
O	-0.5257797	-1.0168423	1.3213406
C	0.2512203	-2.6418423	2.8053406
O	-0.7347797	-3.6318423	2.9163406
C	0.9872203	-2.7868423	1.4923406
O	1.0912203	-4.1318423	1.1183406
C	0.0952203	-2.0278423	0.5423406
N	0.7802203	-1.3858423	-0.5916594
C	0.4702203	-1.6398423	-1.8956594
N	1.2582203	-0.8838423	-2.6896594

C	2.0612203	-0.1328423	-1.9016594
C	3.0422203	0.8101577	-2.2026594
N	3.3132203	1.1041577	-3.4746594
N	3.7202203	1.4431577	-1.1716594
C	3.4222203	1.1311577	0.1453406
N	2.4552203	0.1861577	0.4303406
C	1.7682203	-0.4298423	-0.5706594
H	-2.8537272	0.0623804	-1.5299791
H	0.6056182	2.8489173	-3.3171074
H	-1.4830190	1.4452437	0.0707491
H	-2.3161356	0.0276048	-5.1319068
H	-2.8904499	-1.9274213	-2.8087485
H	-0.8436359	-1.6611036	-4.4801703
H	-4.3913936	-3.0004694	-4.3796391
H	-2.8377291	-2.5203899	-6.1885464
H	-5.3858402	-0.5398615	-5.2848524
H	-6.2086590	-0.0761074	-2.8762449
H	-6.8593706	-1.4968143	-3.7528332
H	5.5503125	2.5881719	3.3435335
H	5.0270430	1.0176401	3.4461248
H	3.6167406	3.4566053	4.0698427
H	2.3426753	0.7746073	4.3542502
H	3.0183313	1.0914988	2.7517230
H	1.5764329	3.0039064	2.3738435
H	1.1359880	3.0625716	4.1220511
H	-2.0495879	1.2824850	4.2505057
H	-0.6298260	1.8971719	5.2083737
H	-1.5152689	2.9846802	4.0644606
H	0.2158898	-0.2922569	4.5386010
H	1.3640312	-0.3283963	3.1476848
H	-1.4784533	-1.4236643	3.1052252
H	0.9476923	-2.6771789	3.6691645
H	-0.3636478	-4.4359193	2.5202414
H	1.9668838	-2.2809158	1.5342628
H	2.0029955	-4.4304727	1.1895089
H	-0.6532187	-2.7119715	0.1084533
H	-0.2834465	-2.3534861	-2.2294608
H	2.8135333	0.6271124	-4.2133378
H	3.9705549	1.6235311	0.9502388
H	5.2301263	1.8252242	6.1070951
H	-6.1673572	-2.1094665	-1.6312662
H	4.0403607	1.7716950	-3.6931241

3E5F (modified)

O	-5.38401720	-2.03719930	-2.24194370
C	-5.95401720	-1.35219930	-3.38494370
C	-4.84701720	-0.98219930	-4.36494370
O	-4.05001720	0.08780070	-3.82494370
C	-3.91301720	-2.15719930	-4.62994370
O	-3.60701720	-2.29619930	-6.03894370
C	-2.57401720	-1.72119930	-4.03194370
O	-1.42101720	-2.15219930	-4.71194370
C	-2.70701720	-0.20019930	-4.06194370
N	-1.87301720	0.60180070	-3.16794370
C	-0.89101720	1.39080070	-3.75794370
O	-0.67501720	1.40580070	-4.96494370
N	-0.17701720	2.16180070	-2.88694370
C	-0.33801720	2.22880070	-1.52494370
O	0.33798280	3.03280070	-0.87794370
C	-1.35101720	1.36780070	-0.99494370
C	-2.06201720	0.59880070	-1.81494370
C	-0.05701720	-2.00819930	0.63705630
C	0.93098280	-2.60019930	1.64305630
C	0.08098280	-2.52719930	2.90905630
C	-0.48701720	-1.11719930	2.74305630
C	0.64898280	-0.13419930	3.02905630
O	4.34998280	1.35680070	6.25405630
C	3.64698280	2.29680070	5.82305630
O	2.74998280	2.89280070	6.45905630
C	3.90098280	2.74980070	4.38205630
N	5.22398280	2.28780070	3.93705630
C	2.83398280	2.14480070	3.46805630
C	1.43098280	2.65480070	3.80705630
Se	0.12998280	1.64880070	2.94105630
C	-1.21801720	1.70680070	4.20005630
O	-0.82701720	-1.02319930	1.34905630
O	-0.97901720	-3.48219930	2.84005630
O	1.15698280	-3.97319930	1.31905630
H	-6.49015560	-0.42400830	-3.07857500
H	-6.68211900	-2.00289140	-3.92647220
H	-5.29857640	-0.65547480	-5.32860710
H	-4.28117720	-3.10534570	-4.18516630
H	-2.58269760	-2.04536250	-2.95569750
H	-0.70262850	-1.51285680	-4.52806020
H	-2.39224330	0.11572880	-5.08313560
H	-1.50491750	1.35382450	0.08865540
H	-2.88303440	-0.05539260	-1.47873800

H	-0.72401550	-2.80470890	0.24962570
H	1.87308140	-2.02032350	1.69139630
H	0.68083560	-2.63673630	3.84398130
H	-1.40537410	-0.96992920	3.34544990
H	1.09643120	-0.32737510	4.02837800
H	1.43396260	-0.19085090	2.22730580
H	3.85782290	3.85630590	4.33779900
H	5.33466200	1.54863820	4.79445390
H	5.96775870	2.99692040	4.02896640
H	2.89644750	1.04197680	3.58740280
H	3.05795490	2.37735750	2.40600630
H	1.30042330	2.64195630	4.91612580
H	1.28241510	3.69487970	3.45278570
H	-0.90546570	1.24611470	5.15802140
H	-1.48100320	2.76823250	4.37576650
H	-2.11296140	1.19620010	3.79808950
H	-0.56240020	-4.28214090	2.44661690
H	2.02954150	-4.24035770	1.66118930
H	-6.12447050	-2.31162550	-1.67229890
H	-4.39489210	-2.67387090	-6.47250700
H	0.53614780	2.76750610	-3.30353460
H	5.28356300	1.83895970	3.00868330
H	0.43763316	-1.58359432	-0.21143155

3E5F (complete)

O	-5.3840172	-2.0371993	-2.2419437
C	-5.9540172	-1.3521993	-3.3849437
C	-4.8470172	-0.9821993	-4.3649437
O	-4.0500172	0.0878007	-3.8249437
C	-3.9130172	-2.1571993	-4.6299437
O	-3.6070172	-2.2961993	-6.0389437
C	-2.5740172	-1.7211993	-4.0319437
O	-1.4210172	-2.1521993	-4.7119437
C	-2.7070172	-0.2001993	-4.0619437
N	-1.8730172	0.6018007	-3.1679437
C	-0.8910172	1.3908007	-3.7579437
O	-0.6750172	1.4058007	-4.9649437
N	-0.1770172	2.1618007	-2.8869437
C	-0.3380172	2.2288007	-1.5249437
O	0.3379828	3.0328007	-0.8779437
C	-1.3510172	1.3678007	-0.9949437
C	-2.0620172	0.5988007	-1.8149437
C	3.3269828	0.8548007	0.1970563
N	2.3429828	0.0168007	0.4730563

C	1.6409828	-0.5601993	-0.5139437
C	-0.0570172	-2.0081993	0.6370563
C	0.9309828	-2.6001993	1.6430563
C	0.0809828	-2.5271993	2.9090563
C	-0.4870172	-1.1171993	2.7430563
C	0.6489828	-0.1341993	3.0290563
O	4.3499828	1.3568007	6.2540563
C	3.6469828	2.2968007	5.8230563
O	2.7499828	2.8928007	6.4590563
C	3.9009828	2.7498007	4.3820563
N	5.2239828	2.2878007	3.9370563
C	2.8339828	2.1448007	3.4680563
C	1.4309828	2.6548007	3.8070563
Se	0.1299828	1.6488007	2.9410563
C	-1.2180172	1.7068007	4.2000563
O	-0.8270172	-1.0231993	1.3490563
O	-0.9790172	-3.4821993	2.8400563
O	1.1569828	-3.9731993	1.3190563
N	0.6279828	-1.4201993	-0.5379437
C	0.3199828	-1.6531993	-1.8139437
N	1.1249828	-0.9521993	-2.6089437
C	1.9549828	-0.2641993	-1.8329437
N	3.6549828	1.1588007	-1.0469437
C	2.9939828	0.6248007	-2.0819437
N	3.2989828	0.9628007	-3.3309437
H	-6.4901556	-0.4240083	-3.0785750
H	-6.6821190	-2.0028914	-3.9264722
H	-5.2985764	-0.6554748	-5.3286071
H	-4.2811772	-3.1053457	-4.1851663
H	-2.5826976	-2.0453625	-2.9556975
H	-0.7026285	-1.5128568	-4.5280602
H	-2.3922433	0.1157288	-5.0831356
H	-1.5049175	1.3538245	0.0886554
H	-2.8830344	-0.0553926	-1.4787380
H	3.9081137	1.2946665	1.0232391
H	-0.7240155	-2.8047089	0.2496257
H	1.8730814	-2.0203235	1.6913963
H	0.6808356	-2.6367363	3.8439813
H	-1.4053741	-0.9699292	3.3454499
H	1.0964312	-0.3273751	4.0283780
H	1.4339626	-0.1908509	2.2273058
H	3.8578229	3.8563059	4.3377990
H	5.3346620	1.5486382	4.7944539
H	5.9677587	2.9969204	4.0289664

H	2.8964475	1.0419768	3.5874028
H	3.0579549	2.3773575	2.4060063
H	1.3004233	2.6419563	4.9161258
H	1.2824151	3.6948797	3.4527857
H	-0.9054657	1.2461147	5.1580214
H	-1.4810032	2.7682325	4.3757665
H	-2.1129614	1.1962001	3.7980895
H	-0.5624002	-4.2821409	2.4466169
H	2.0295415	-4.2403577	1.6611893
H	-0.4721020	-2.3370513	-2.1434725
H	4.0710928	1.6044583	-3.5115478
H	-6.1244705	-2.3116255	-1.6722989
H	-4.3948921	-2.6738709	-6.4725070
H	0.5361478	2.7675061	-3.3035346
H	2.7938191	0.5404387	-4.1104764
H	5.2835630	1.8389597	3.0086833

3GX5

O	-5.4607797	-1.9558423	-2.2656594
C	-5.9557797	-1.0738423	-3.2766594
C	-4.8957797	-0.8658423	-4.3526594
O	-4.0147797	0.1931577	-3.9036594
C	-3.9627797	-2.0318423	-4.6876594
O	-3.6667797	-2.0278423	-6.1056594
C	-2.7087797	-1.6898423	-3.8716594
O	-1.5427797	-2.3188423	-4.3696594
C	-2.6717797	-0.1828423	-4.1096594
N	-1.8097797	0.6911577	-3.2196594
C	-0.8337797	1.4941577	-3.8056594
O	-0.6087797	1.5361577	-5.0096594
N	-0.1157797	2.2561577	-2.9186594
C	-0.2847797	2.3021577	-1.5366594
O	0.4342203	3.0361577	-0.8626594
C	-1.3177797	1.4571577	-1.0026594
C	-2.0287797	0.7051577	-1.8496594
N	5.0542203	1.9151577	3.9183406
C	3.7052203	2.3941577	4.3533406
C	3.5222203	2.3961577	5.8843406
O	4.4402203	2.0551577	6.6443406
O	2.4542203	2.7521577	6.4113406
C	2.6262203	1.5731577	3.6563406
C	1.4002203	2.4041577	3.2803406
S	-0.0997797	1.4611577	2.8943406
C	-1.1817797	1.9561577	4.2593406

C	0.3142203	-0.2228423	3.4463406
C	-0.4577797	-1.3088423	2.7053406
O	-0.5257797	-1.0168423	1.3213406
C	0.2512203	-2.6418423	2.8053406
O	-0.7347797	-3.6318423	2.9163406
C	0.9872203	-2.7868423	1.4923406
O	1.0912203	-4.1318423	1.1183406
C	0.0952203	-2.0278423	0.5423406
N	0.7802203	-1.3858423	-0.5916594
C	0.4702203	-1.6398423	-1.8956594
N	1.2582203	-0.8838423	-2.6896594
C	2.0612203	-0.1328423	-1.9016594
C	3.0422203	0.8101577	-2.2026594
N	3.3132203	1.1041577	-3.4746594
N	3.7202203	1.4431577	-1.1716594
C	3.4222203	1.1311577	0.1453406
N	2.4552203	0.1861577	0.4303406
C	1.7682203	-0.4298423	-0.5706594
H	-2.8537272	0.0623804	-1.5299791
H	0.6056182	2.8489173	-3.3171074
H	-1.4830190	1.4452437	0.0707491
H	-2.3161356	0.0276048	-5.1319068
H	-2.8904499	-1.9274213	-2.8087485
H	-0.8436359	-1.6611036	-4.4801703
H	-4.3913936	-3.0004694	-4.3796391
H	-2.8377291	-2.5203899	-6.1885464
H	-5.3858402	-0.5398615	-5.2848524
H	-6.2086590	-0.0761074	-2.8762449
H	-6.8593706	-1.4968143	-3.7528332
H	5.5503125	2.5881719	3.3435335
H	5.0270430	1.0176401	3.4461248
H	3.6167406	3.4566053	4.0698427
H	2.3426753	0.7746073	4.3542502
H	3.0183313	1.0914988	2.7517230
H	1.5764329	3.0039064	2.3738435
H	1.1359880	3.0625716	4.1220511
H	-2.0495879	1.2824850	4.2505057
H	-0.6298260	1.8971719	5.2083737
H	-1.5152689	2.9846802	4.0644606
H	0.2158898	-0.2922569	4.5386010
H	1.3640312	-0.3283963	3.1476848
H	-1.4784533	-1.4236643	3.1052252
H	0.9476923	-2.6771789	3.6691645
H	-0.3636478	-4.4359193	2.5202414

H	1.9668838	-2.2809158	1.5342628
H	2.0029955	-4.4304727	1.1895089
H	-0.6532187	-2.7119715	0.1084533
H	-0.2834465	-2.3534861	-2.2294608
H	2.8135333	0.6271124	-4.2133378
H	3.9705549	1.6235311	0.9502388
H	5.2301263	1.8252242	6.1070951
H	-6.1673572	-2.1094665	-1.6312662
H	4.0403607	1.7716950	-3.6931241

3GX6

O	-2.4746182	-5.9259148	3.2649112
C	-3.3006182	-4.7749148	3.1629112
C	-2.9396182	-3.9369148	1.9479112
O	-1.7046182	-3.2149148	2.1949112
C	-2.6766182	-4.7079148	0.6609112
O	-3.8836182	-5.0189148	-0.0210888
C	-1.8796182	-3.6589148	-0.0960888
O	-2.7046182	-2.6059148	-0.5500888
C	-0.9466182	-3.1689148	1.0029112
N	0.2393818	-4.0499148	1.1559112
C	1.3423818	-3.8289148	0.3609112
O	1.3973818	-2.9339148	-0.4610888
N	2.3883818	-4.6939148	0.5659112
C	2.4373818	-5.7389148	1.4639112
O	3.4423818	-6.4339148	1.5359112
C	1.2493818	-5.9119148	2.2549112
C	0.2183818	-5.0789148	2.0749112
N	1.4913818	3.9980852	-2.9200888
C	1.2863818	3.1780852	-4.1110888
C	-0.1136182	3.3580852	-4.6920888
O	-0.6006182	2.5180852	-5.4520888
O	-0.7836182	4.3530852	-4.4150888
C	1.5823818	1.7100852	-3.8190888
C	1.9073818	1.4400852	-2.3580888
S	1.6583818	-0.2819148	-1.8590888
C	0.7603818	-0.9139148	-3.2980888
C	0.3153818	-0.1009148	-0.6540888
C	0.8843818	0.4070852	0.6629112
O	1.4773818	1.6630852	0.4339112
C	-0.1286182	0.6590852	1.7639112
O	-0.2156182	-0.4489148	2.6319112
C	0.4533818	1.8500852	2.4979112
O	0.9943818	1.4010852	3.7159112

C	1.5673818	2.4070852	1.6219112
N	1.2783818	3.8190852	1.3199112
C	2.0093818	4.8980852	1.7419112
N	1.4403818	6.0270852	1.2579112
C	0.3483818	5.6870852	0.5399112
C	-0.5836182	6.4490852	-0.1540888
N	-0.4736182	7.7750852	-0.1810888
N	-1.6206182	5.8250852	-0.8130888
C	-1.7176182	4.4480852	-0.7750888
N	-0.7856182	3.6960852	-0.0850888
C	0.2393818	4.3020852	0.5599112
H	-3.2227332	-4.1186973	4.0610628
H	-4.3738114	-5.0604526	3.0475295
H	-3.7613000	-3.2082070	1.7698558
H	-2.0754129	-5.6162879	0.8736397
H	-1.2905851	-4.1032059	-0.9371922
H	-3.5514673	-3.0396150	-0.8000061
H	-0.5762143	-2.1399025	0.8634296
H	1.2262579	-6.7250167	2.9888776
H	-0.6970234	-5.1710047	2.6738783
H	2.4232685	4.4186962	-2.7765183
H	1.1444858	3.5849451	-2.0351716
H	1.9838687	3.5707141	-4.8786755
H	0.6704267	1.2077678	-4.1926822
H	2.4121719	1.3444236	-4.4564438
H	1.2441899	1.9696321	-1.6526178
H	2.9486136	1.6897463	-2.0740658
H	0.5369803	-1.9665376	-3.0320034
H	-0.1728720	-0.3522648	-3.4798301
H	1.4356711	-0.8963975	-4.1698634
H	-0.1008647	-1.1181027	-0.5439479
H	-0.4388289	0.6022592	-1.0660997
H	1.6282258	-0.3311587	1.0497649
H	-1.1242669	0.9030978	1.3375629
H	0.3644983	-0.2100174	3.3933517
H	-0.3171701	2.6273591	2.6623333
H	0.5245021	1.8060464	4.4670376
H	2.5568006	2.3381907	2.1231721
H	2.9089655	4.8263479	2.3646774
H	0.2706973	8.2317485	0.3453706
H	-2.5309289	3.9464882	-1.3176738
H	-2.6729411	-6.3793363	4.1037634
H	-3.7261723	-5.7923945	-0.5920266
H	0.7265808	4.7550168	-3.1403708

H	-1.1793436	8.3300500	-0.6665632
H	3.2194329	-4.5507852	-0.0164483

3IQN

O	-2.9335847	-6.1307061	3.1077699
C	-3.5445847	-4.8577061	3.1767699
C	-3.1495847	-4.0187061	1.9907699
O	-1.8615847	-3.4017061	2.2617699
C	-2.9275847	-4.7537061	0.6817699
O	-4.1255847	-5.0337061	-0.0392301
C	-2.0295847	-3.7747061	-0.0562301
O	-2.7735847	-2.6557061	-0.4942301
C	-1.1065847	-3.3177061	1.0677699
N	0.0884153	-4.1627061	1.2167699
C	1.2254153	-3.8327061	0.4847699
O	1.2694153	-2.8797061	-0.2842301
N	2.3104153	-4.6547061	0.6907699
C	2.3764153	-5.7507061	1.5307699
O	3.4244153	-6.4017061	1.6147699
C	1.1654153	-6.0217061	2.2407699
C	0.0904153	-5.2407061	2.0607699
N	1.0404153	4.1512939	-3.0312301
C	0.8954153	3.2712939	-4.1982301
C	-0.3815847	3.6212939	-4.9012301
O	-0.7235847	2.9942939	-5.8912301
O	-1.0825847	4.5232939	-4.4702301
C	0.8484153	1.8012939	-3.7552301
C	1.9574153	1.5562939	-2.7362301
S	1.7734153	-0.0107061	-1.8262301
C	0.7974153	-1.0717061	-2.9362301
C	0.5534153	0.1612939	-0.4742301
C	1.4084153	0.5282939	0.7137699
O	1.5864153	1.9002939	0.5847699
C	0.8864153	0.4272939	2.1477699
O	1.1444153	-0.8197061	2.7267699
C	1.7254153	1.6322939	2.7917699
O	3.1464153	1.5272939	2.6207699
C	1.0434153	2.5482939	1.8017699
N	1.1544153	3.9362939	1.3917699
C	2.0454153	4.9812939	1.3857699
N	1.5354153	6.0442939	0.8037699
C	0.2894153	5.7652939	0.3737699
C	-0.7295847	6.4892939	-0.3042301

N	-0.5535847	7.7952939	-0.6662301
N	-1.8715847	5.8752939	-0.5862301
C	-2.0665847	4.5892939	-0.2902301
N	-1.1755847	3.8852939	0.4067699
C	0.0144153	4.4072939	0.7327699
H	-3.2659679	-4.2924131	4.0965900
H	-4.6573422	-4.9509337	3.1686373
H	-3.9085123	-3.2186455	1.8394571
H	-2.3747714	-5.6931455	0.8992425
H	-1.4569340	-4.2528248	-0.8878458
H	-3.6287514	-3.0234652	-0.8090453
H	-0.7495313	-2.2803099	0.9119602
H	1.1492515	-6.8802635	2.9216949
H	-0.8515009	-5.4241640	2.5983793
H	1.9540348	4.5870602	-2.8857047
H	0.6773311	3.7880713	-2.1422912
H	1.7363454	3.4453656	-4.8992888
H	-0.1427230	1.6276621	-3.2814653
H	0.9086400	1.1387048	-4.6372653
H	2.0030237	2.3083515	-1.9243153
H	2.9647939	1.4850337	-3.1956561
H	0.7501389	-2.0412050	-2.4020231
H	-0.2098761	-0.6476239	-3.0942929
H	1.3409982	-1.1831405	-3.8920515
H	0.1302591	-0.8626900	-0.4137318
H	-0.2166736	0.9279525	-0.6902209
H	2.3811572	-0.0175353	0.6876579
H	-0.2000923	0.5932656	2.2597409
H	2.1107899	-0.9695444	2.6871933
H	1.4213830	1.7735115	3.8494877
H	3.5338798	1.4599212	3.5120738
H	-0.0579793	2.4245427	1.8718552
H	3.0442777	4.8959506	1.8319096
H	0.2681628	8.3018170	-0.3402285
H	-3.0198690	4.1124229	-0.5720661
H	-0.5874862	4.9421496	-3.7010811
H	-1.3462442	8.3041594	-1.0574969
H	-3.0652301	-6.5907930	3.9560032
H	-4.0036886	-5.8734225	-0.5159753
H	3.1547513	-4.4362165	0.1523752

3IQR

O	-2.8128449	-6.2016557	3.3170794

C	-3.6238449	-5.0356557	3.3060794
C	-3.2328449	-4.1266557	2.1670794
O	-1.9798449	-3.4566557	2.4780794
C	-2.9568449	-4.8186557	0.8480794
O	-4.1228449	-5.1426557	0.1070794
C	-2.0828449	-3.8006557	0.1430794
O	-2.8768449	-2.7476557	-0.3619206
C	-1.2128449	-3.3026557	1.2970794
N	0.0001551	-4.1226557	1.4300794
C	1.1391551	-3.7336557	0.7340794
O	1.1841551	-2.7376557	0.0270794
N	2.2271551	-4.5606557	0.8970794
C	2.2941551	-5.7076557	1.6650794
O	3.3471551	-6.3486557	1.7120794
C	1.0841551	-6.0356557	2.3480794
C	0.0071551	-5.2516557	2.2120794
N	1.0181551	4.2793443	-2.8049206
C	1.0471551	3.4123443	-3.9819206
C	-0.1378449	3.7173443	-4.8519206
O	-0.3268449	3.0723443	-5.8779206
O	-0.9348449	4.5823443	-4.5119206
C	0.9931551	1.9443443	-3.5609206
C	1.9921551	1.7173443	-2.4409206
S	1.9961551	0.0153443	-1.7929206
C	1.1701551	-0.9636557	-3.0819206
C	0.4441551	-0.0516557	-0.7109206
C	0.4981551	0.5243443	0.7660794
O	0.1381551	1.8713443	0.9740794
C	1.6391551	0.2233443	1.6530794
O	2.4091551	-0.8916557	1.2020794
C	2.3561551	1.6493443	1.5200794
O	3.3721551	1.7833443	2.4680794
C	1.1741551	2.5763443	1.7970794
N	1.1061551	4.0283443	1.3450794
C	1.8951551	5.1413443	1.4280794
N	1.2991551	6.1743443	0.8950794
C	0.0981551	5.8003443	0.4200794
C	-0.9728449	6.4573443	-0.2469206
N	-0.9118449	7.8053443	-0.5369206
N	-2.0568449	5.7353443	-0.5929206
C	-2.1298449	4.4223443	-0.3589206
N	-1.1748449	3.7883443	0.3150794
C	-0.0508449	4.4163443	0.6950794
H	-0.9228229	-5.4661901	2.7430008

H	3.0692913	-4.2992038	0.3939671
H	1.0704887	-6.9237244	2.9766636
H	-0.9048320	-2.2575752	1.1604853
H	-1.4567977	-4.2484493	-0.6521460
H	-3.6943181	-3.1656934	-0.6741573
H	-2.3758558	-5.7311524	1.0588236
H	-4.0218119	-3.3672370	2.0306473
H	-3.5360294	-4.4636841	4.2468969
H	-4.6886352	-5.2986022	3.1643549
H	0.5324457	3.9173040	-1.9882704
H	1.8764581	4.7501386	-2.5469140
H	1.9573663	3.6134709	-4.5671340
H	1.1806658	1.3153055	-4.4401252
H	-0.0287002	1.7234236	-3.2085896
H	1.7953656	2.3457327	-1.5623891
H	3.0278030	1.9015367	-2.7683200
H	1.1129576	-1.9727373	-2.6487886
H	1.7883859	-0.9838193	-3.9887349
H	0.1714532	-0.5641497	-3.2859401
H	0.3015689	-1.1366318	-0.6224342
H	-0.3541909	0.4593839	-1.2662844
H	-0.3834105	-0.0414252	1.1376014
H	1.3171863	0.1097312	2.7024926
H	3.1856152	-0.9344579	1.7766655
H	2.7139186	1.7635001	0.4798075
H	4.0417418	2.4024463	2.1610267
H	0.9032233	2.5437135	2.8642240
H	2.8712348	5.1413357	1.9131398
H	-0.1766488	8.3589700	-0.1207941
H	-3.0207307	3.8720211	-0.6759048
H	-3.0022950	-6.7055211	4.1149155
H	-3.9923968	-5.9953279	-0.3179807
H	-0.5906417	5.0259543	-3.7020679
H	-1.7524262	8.2545465	-0.8744421

3V7E

O	-1.2613293	-4.5638374	4.2451284
C	-2.2073293	-3.6388374	3.7141284
C	-1.6853293	-3.0088374	2.4441284
O	-0.5953293	-2.0908374	2.7371284
C	-1.0683293	-3.9538374	1.4361284
O	-2.0523293	-4.6168374	0.6741284
C	-0.2793293	-2.9818374	0.5801284
O	-1.1513293	-2.2028374	-0.2128716

C	0.3306707	-2.0928374	1.6581284
N	1.6166707	-2.6028374	2.1561284
C	2.7696707	-2.1288374	1.5621284
O	2.7616707	-1.3658374	0.6181284
N	3.9356707	-2.5848374	2.1131284
C	4.0646707	-3.4608374	3.1561284
O	5.1886707	-3.7738374	3.5391284
C	2.8296707	-3.9328374	3.6981284
C	1.6776707	-3.5018374	3.1911284
N	-2.7153293	1.8171626	-0.3258716
C	-2.3223293	2.9521626	0.5091284
C	-2.8063293	4.3381626	0.0251284
O	-3.3203293	5.1031626	0.8431284
O	-2.7033293	4.7271626	-1.1478716
C	-0.8073293	2.9771626	0.6761284
C	-0.2703293	1.8081626	1.5041284
S	1.5506707	1.8931626	1.6841284
C	1.6736707	3.4241626	2.6581284
C	2.2056707	2.3421626	0.0581284
C	2.2206707	1.1591626	-0.9078716
O	0.9476707	0.6121626	-1.1148716
C	2.7196707	1.4921626	-2.2988716
O	4.1276707	1.4761626	-2.3228716
C	2.0696707	0.4231626	-3.1508716
O	3.0456707	-0.5638374	-3.4188716
C	0.9056707	-0.1258374	-2.3158716
N	-0.4393293	0.1241626	-2.9148716
C	-1.4453293	-0.8118374	-3.0708716
N	-2.5263293	-0.2298374	-3.6448716
C	-2.2343293	1.0761626	-3.8598716
C	-2.9563293	2.1481626	-4.4178716
N	-4.1943293	1.9871626	-4.8678716
N	-2.3623293	3.4021626	-4.4988716
C	-1.0653293	3.6151626	-4.0428716
N	-0.3653293	2.5481626	-3.5008716
C	-0.9243293	1.3081626	-3.4048716
H	-2.4447173	-2.8207488	4.4337322
H	-3.1672806	-4.1435899	3.4483030
H	-2.5252228	-2.4571412	1.9623953
H	-0.4006113	-4.6686250	1.9610500
H	0.5114672	-3.4701718	-0.0351009
H	-1.8736312	-2.8225209	-0.4691039
H	0.5216030	-1.0757821	1.2566444
H	2.8694827	-4.6395605	4.5347778

H	0.7094741	-3.7986276	3.6226698
H	-2.1741559	0.9339580	-0.2291131
H	-3.7216064	1.5965591	-0.2314432
H	-2.8052867	2.8111685	1.4984427
H	-0.5926961	3.9451710	1.1693120
H	-0.3596379	3.0024692	-0.3398814
H	-0.6491248	1.7838808	2.5460690
H	-0.4371263	0.8144914	1.0397705
H	2.7453924	3.5953888	2.8728635
H	1.1308812	3.2419208	3.6045689
H	1.2447628	4.2839441	2.1115687
H	3.2595713	2.6357864	0.2442232
H	1.6505789	3.2183458	-0.3336819
H	2.8982088	0.3831558	-0.4886204
H	2.3316708	2.4840627	-2.6191951
H	4.3534124	0.5922525	-2.6969250
H	1.6799873	0.8702533	-4.0872983
H	3.0523893	-0.7709400	-4.3697897
H	1.0088248	-1.2177043	-2.1437437
H	-1.3418978	-1.8516533	-2.7453794
H	-4.6183662	1.0608324	-4.8841318
H	-1.5778811	-4.8734855	5.1123850
H	-4.6750898	2.7824083	-5.2907050
H	-0.6148242	4.6146816	-4.0948663
H	-2.5971656	2.1594878	-1.3272353
H	4.8035278	-2.2517906	1.6805474
H	-1.6971098	-5.4688110	0.3629851

4AOB

O	2.7893257	6.0768815	3.4284202
C	3.6293257	4.9708815	3.2034202
C	2.9593257	4.0098815	2.2634202
O	1.6303257	3.6768815	2.7464202
C	2.6643257	4.4728815	0.8424202
O	3.7903257	4.5548815	0.0144202
C	1.7683257	3.3398815	0.4174202
O	2.5543257	2.1828815	0.7014202
C	0.8153257	3.3118815	1.6164202
N	-0.3206743	4.2458815	1.5244202
C	-1.3176743	3.9408815	0.6224202
O	-1.2796743	2.9468815	-0.1025798
N	-2.3696743	4.8348815	0.6034202
C	-2.5296743	5.9668815	1.3844202
O	-3.5266743	6.6728815	1.2334202

C	-1.4626743	6.2078815	2.3034202
C	-0.4136743	5.3638815	2.3394202
N	-1.6456743	-4.2941185	-3.0985798
C	-1.1376743	-3.2421185	-4.0115798
C	0.0743257	-3.8051185	-4.7425798
O	0.3573257	-5.0391185	-4.8995798
O	0.8793257	-2.9761185	-5.1705798
C	-0.8226743	-1.9891185	-3.1915798
C	-2.0706743	-1.2001185	-2.8185798
S	-2.0196743	0.4108815	-1.9475798
C	-1.1936743	1.3768815	-3.2105798
C	-0.6906743	0.0228815	-0.7645798
C	-1.2416743	-0.4951185	0.5354202
O	-1.9536743	-1.7461185	0.3774202
C	-0.1996743	-0.7141185	1.6084202
O	-0.2046743	0.3698815	2.4314202
C	-0.8326743	-1.8561185	2.3684202
O	-1.7256743	-1.2811185	3.2154202
C	-1.5976743	-2.7091185	1.3324202
N	-0.5286743	-3.5251185	0.6954202
C	0.7943257	-3.1501185	0.2224202
N	1.5833257	-4.1711185	-0.2745798
C	0.7613257	-5.1801185	-0.1115798
C	1.0473257	-6.4491185	-0.4305798
N	2.2443257	-6.6541185	-0.9405798
N	0.0803257	-7.4021185	-0.1955798
C	-1.1456743	-6.9331185	0.3854202
N	-1.4846743	-5.6071185	0.7524202
C	-0.5356743	-4.7981185	0.4904202
H	3.8655873	4.4261468	4.1494919
H	4.6148824	5.2466198	2.7432516
H	3.6047618	3.1122703	2.2344645
H	2.1075463	5.4410555	0.8830232
H	1.3036775	3.3872777	-0.5882178
H	3.3711872	2.2664826	0.1664329
H	0.4079881	2.2918456	1.7693540
H	-1.5191626	7.0898762	2.9509465
H	0.4569680	5.5006855	2.9996352
H	-2.6642495	-4.4604341	-3.0580713
H	-1.2728021	-4.2270336	-2.1327712
H	-1.9250601	-3.0470670	-4.7693490
H	-0.2387017	-2.2967542	-2.2966356
H	-0.1207544	-1.4231806	-3.8434399
H	-2.7182521	-1.7903011	-2.1334527

H	-2.6831835	-0.9812206	-3.7198702
H	-0.9647043	2.3593820	-2.7530655
H	-0.2744300	0.8690604	-3.5568735
H	-1.9125241	1.5059346	-4.0417008
H	-0.2241220	1.0067344	-0.5407866
H	0.0395180	-0.6459900	-1.2546456
H	-1.9559076	0.2578671	0.9328727
H	0.8299117	-0.8831529	1.2585779
H	-0.9150639	0.2197025	3.0982356
H	-0.0688475	-2.4697128	2.8989226
H	-1.9188540	-1.8480574	3.9830754
H	-2.4251655	-3.3658336	1.6554107
H	2.8417616	-5.8482545	-1.1255784
H	-3.1226125	4.6375983	-0.0628177
H	3.2022229	6.6668856	4.0836831
H	4.3681861	5.2732047	0.3296601
H	-1.9005607	-7.7117018	0.5670095
H	2.5299692	-7.5776919	-1.2667710
H	1.1344720	-2.1172634	0.2074629
H	-1.1284877	-5.1508895	-3.5662719

4KQY

O	-4.83266330	-5.94118700	-1.23143770
C	-5.54066330	-4.69918700	-1.15343770
C	-4.54166330	-3.59118700	-1.18943770
O	-3.49966330	-3.88118700	-0.23443770
C	-3.76166330	-3.50818700	-2.48243770
O	-4.55766330	-3.03218700	-3.55043770
C	-2.56066330	-2.68518700	-2.04943770
O	-2.76966330	-1.31518700	-1.78543770
C	-2.28566330	-3.30818700	-0.68743770
N	-1.26666330	-4.35718700	-0.73043770
C	0.02833670	-3.96118700	-0.97743770
O	0.33233670	-2.80218700	-1.20543770
N	0.95333670	-4.97318700	-0.95243770
C	0.71933670	-6.30918700	-0.71143770
O	1.65833670	-7.10518700	-0.74643770
C	-0.64666330	-6.63418700	-0.45143770
C	-1.57166330	-5.67118700	-0.47443770
N	3.62633670	4.52281300	-0.30343770
C	3.12633670	4.08681300	-1.59343770
C	2.22133670	5.16381300	-2.10743770
O	0.99433670	5.08881300	-1.88143770
O	2.71333670	6.13681300	-2.72743770

C	2.35833670	2.76981300	-1.45243770
C	3.31833670	1.56881300	-1.30243770
S	2.58333670	0.06481300	-1.18843770
C	2.50733670	-0.62718700	-2.69643770
C	1.03833670	0.23381300	-0.53043770
C	0.96533670	-0.05118700	0.98456230
O	1.98133670	0.63081300	1.73856230
C	-0.30866330	0.45881300	1.63856230
O	-1.51966330	-0.28718700	1.40256230
C	0.11733670	0.48881300	3.08556230
O	0.21733670	-0.83818700	3.59356230
C	1.49233670	1.08281300	3.00356230
N	1.46933670	2.57181300	3.13556230
C	2.26933670	3.21181300	3.98756230
N	2.04733670	4.53281300	3.94756230
C	1.11333670	4.78081300	3.05756230
C	0.46233670	6.01181300	2.55256230
N	0.79533670	7.26781300	3.01556230
N	-0.49866330	5.80881300	1.59756230
C	-0.83666330	4.57381300	1.15356230
N	-0.23966330	3.45381300	1.59456230
C	0.72133670	3.48481300	2.52456230
H	-6.14266570	-4.64104890	-0.21484160
H	-6.23207040	-4.54211330	-2.01419990
H	-5.02333680	-2.60800050	-0.98347540
H	-3.36823610	-4.53433980	-2.69069660
H	-1.70103570	-2.83199310	-2.74536450
H	-2.08226220	-1.02835800	-1.15343350
H	-1.91825680	-2.53074270	0.02080190
H	-0.90189260	-7.68211260	-0.25818280
H	-2.64545140	-5.85595160	-0.32140470
H	4.27959740	3.87031230	0.17040080
H	2.81173210	4.68865420	0.33115670
H	4.00285230	3.99037290	-2.26563590
H	1.64082340	2.87003790	-0.61186190
H	1.73432150	2.66030450	-2.35973400
H	3.95897080	1.70645410	-0.39647900
H	3.98415940	1.46500750	-2.17964070
H	2.48745130	-1.69258996	-2.59937530
H	1.62006841	-0.29790224	-3.19566363
H	0.52760120	1.19341020	-0.76414940
H	0.46025390	-0.57399070	-1.01377340
H	1.08695900	-1.14838980	1.14027710
H	-0.50565130	1.50157590	1.30645510

H	-1.41756070	-1.10835510	1.93063120
H	-0.57348060	1.10861540	3.69889640
H	-0.08124550	-0.83752690	4.51878450
H	2.15949770	0.72238380	3.81239290
H	2.99463180	2.69957580	4.63145060
H	1.42150270	7.34963200	3.81397320
H	-1.60370940	4.49171940	0.36937450
H	0.25062090	8.06612940	2.68975910
H	4.08585120	5.45078620	-0.43769800
H	-3.96493620	-2.79625860	-4.28660650
H	-5.47061810	-6.63585890	-1.46998580
H	1.91999590	-4.70066990	-1.15701440
H	3.36553001	-0.33686598	-3.26575127

4L81

O	5.6850934	-3.1192739	-2.9976078
C	4.3790934	-3.6222739	-3.2356078
C	3.3500934	-2.5592739	-2.9386078
O	3.5060934	-2.1282739	-1.5616078
C	3.4950934	-1.2562739	-3.6956078
O	2.9950934	-1.3072739	-5.0076078
C	2.7220934	-0.2882739	-2.8156078
O	1.3150934	-0.4072739	-2.8616078
C	3.1940934	-0.7432739	-1.4486078
N	4.4040934	-0.0182739	-1.0456078
C	4.2510934	1.2627261	-0.5436078
O	3.1610934	1.8277261	-0.4646078
N	5.4130934	1.8587261	-0.1336078
C	6.6670934	1.3197261	-0.1736078
O	7.5830934	1.8997261	0.3903922
C	6.7390934	0.0197261	-0.7376078
C	5.6360934	-0.5892739	-1.1436078
N	-4.7249066	2.3067261	1.3683922
C	-4.3899066	2.9567261	0.0963922
C	-5.3269066	2.4627261	-0.9856078
O	-5.1709066	2.8197261	-2.1436078
O	-6.2219066	1.6807261	-0.7166078
C	-2.9449066	2.6537261	-0.3226078
C	-1.9919066	2.8347261	0.8503922
S	-0.2259066	2.6777261	0.3813922
C	-0.2129066	3.0327261	-1.4056078
C	-0.2189066	0.8877261	0.3133922
C	0.2950934	0.2357261	1.5693922
O	-0.6309066	0.5287261	2.6293922

C	0.1500934	-1.2022739	1.2713922
O	1.3990934	-1.7272739	0.8243922
C	-0.1959066	-1.7762739	2.6103922
O	1.0040934	-2.0942739	3.3453922
C	-0.9969066	-0.6692739	3.3593922
N	-2.4859066	-0.7412739	3.2963922
C	-3.3149066	-0.3362739	4.3163922
N	-4.5669066	-0.5132739	4.0153922
C	-4.6489066	-1.0292739	2.7833922
C	-5.7239066	-1.4192739	1.9303922
N	-7.0369066	-1.3032739	2.3393922
N	-5.4279066	-1.9002739	0.7043922
C	-4.1479066	-1.9972739	0.2683922
N	-3.1019066	-1.6892739	1.0633922
C	-3.3009066	-1.1882739	2.2933922
H	4.1580758	-4.5101650	-2.5969287
H	4.2298920	-3.9271655	-4.2999876
H	2.3312803	-2.9817671	-3.1033207
H	4.5724174	-0.9754087	-3.6754867
H	3.0306044	0.7664579	-3.0086129
H	1.0928969	-0.6187637	-3.7941192
H	2.4312931	-0.6053567	-0.6608455
H	7.7228376	-0.4597826	-0.7962874
H	5.6431093	-1.6103186	-1.5525645
H	-4.7040449	2.9001856	2.2009795
H	-6.2211003	1.5421670	0.2774987
H	-4.5391291	4.0513327	0.1977637
H	-2.9005483	1.6091682	-0.7011662
H	-2.7100667	3.3098220	-1.1794903
H	-2.0951905	2.0888936	1.6622907
H	-2.0928138	3.8442547	1.3014426
H	-0.8617372	2.3259947	-1.9499412
H	-0.5073610	4.0856823	-1.5686018
H	0.8494744	2.8832662	-1.6882027
H	-1.2448949	0.5383620	0.0873807
H	0.4484555	0.6111167	-0.5325121
H	1.3361517	0.5206978	1.8273950
H	-0.6616895	-1.4338599	0.5533845
H	1.8633053	-1.9967093	1.6509132
H	-0.8251299	-2.6841598	2.4930006
H	0.8958350	-2.9732217	3.7503949
H	-0.7036391	-0.5985278	4.4247594
H	-2.9160833	0.0670619	5.2573270
H	-7.2346451	-1.0722429	3.3121409

H	-3.9561223	-2.3799076	-0.7459759
H	-4.2198925	1.4318704	1.5542237
H	-7.7675006	-1.6981756	1.7465534
H	3.4662193	-0.6496528	-5.5487414
H	6.3324108	-3.8094662	-3.2260388
H	5.3230758	2.7880533	0.2888565

4OQU

O	-58.6860000	35.0320000	-16.3730000
C	-58.4270000	33.6400000	-16.3380000
C	-56.9630000	33.3560000	-16.5280000
O	-56.5500000	33.7610000	-17.8500000
C	-56.0320000	34.1170000	-15.6170000
O	-55.9600000	33.5400000	-14.3350000
C	-54.7190000	34.0840000	-16.3830000
O	-54.0750000	32.8270000	-16.2370000
C	-55.2120000	34.2090000	-17.8180000
N	-55.1950000	35.6050000	-18.2820000
C	-53.9970000	36.1180000	-18.7420000
O	-52.9740000	35.4460000	-18.7720000
N	-54.0680000	37.4320000	-19.1610000
C	-55.2080000	38.2280000	-19.1580000
O	-55.1710000	39.3970000	-19.5530000
C	-56.3930000	37.5960000	-18.6630000
C	-56.3530000	36.3340000	-18.2510000
N	-48.5080000	28.5210000	-19.7680000
C	-48.1730000	29.6240000	-18.9200000
C	-47.7360000	29.0510000	-17.6210000
O	-47.2850000	29.8070000	-16.7130000
O	-47.8100000	27.8040000	-17.4440000
C	-49.3590000	30.5560000	-18.7530000
C	-48.8530000	31.9920000	-18.7690000
S	-50.1430000	33.1410000	-19.2790000
C	-49.8290000	34.6710000	-18.4670000
C	-51.7960000	32.4800000	-19.0110000
C	-52.3570000	32.0090000	-20.3570000
O	-51.4510000	31.2020000	-21.1260000
C	-53.6600000	31.2920000	-20.3180000
O	-54.6990000	32.1960000	-20.4140000
C	-53.6390000	30.4450000	-21.5250000
O	-54.4950000	30.9980000	-22.4540000
C	-52.2460000	30.4530000	-22.0120000
N	-51.6770000	29.1180000	-22.0540000
C	-50.9430000	28.5770000	-23.0950000

N	-50.5330000	27.3280000	-22.7990000
C	-50.9920000	27.0110000	-21.5370000
C	-50.8650000	25.8640000	-20.7160000
N	-50.1440000	24.7090000	-21.1760000
N	-51.4430000	25.9010000	-19.5020000
C	-52.1160000	26.9840000	-19.0700000
N	-52.2560000	28.0950000	-19.8060000
C	-51.7200000	28.1430000	-21.0320000
H	-58.7088925	33.1885331	-15.3555507
H	-58.9916697	33.0905847	-17.1282107
H	-56.7954719	32.2618792	-16.3913464
H	-56.3994399	35.1678493	-15.5682155
H	-54.0357866	34.9235969	-16.1170489
H	-54.2750365	32.5399893	-15.3180679
H	-54.5745154	33.6258019	-18.5109422
H	-57.3117844	38.1925944	-18.6416102
H	-57.2359289	35.8106662	-17.8586862
H	-48.2204031	27.4588193	-18.3057821
H	-49.3793448	28.6233628	-20.2994241
H	-47.2978204	30.1970392	-19.3017642
H	-49.9163251	30.3483302	-17.8192282
H	-50.0529385	30.3965013	-19.6028235
H	-48.4343366	32.2781285	-17.7813932
H	-48.0745737	32.1422870	-19.5424862
H	-49.7335963	34.5693711	-17.3680434
H	-50.6788290	35.3401778	-18.7168291
H	-48.8903564	35.0543621	-18.9105318
H	-51.7859348	31.6997129	-18.2248132
H	-52.4006980	33.3404545	-18.6483734
H	-52.5525712	32.9453861	-20.9409011
H	-53.7251047	30.6600694	-19.4047294
H	-55.3858555	32.0316242	-19.7392193
H	-53.9447603	29.4023788	-21.2753731
H	-55.0461403	31.6548061	-21.9736998
H	-52.2141024	30.8687292	-23.0431983
H	-50.7536205	29.1213621	-24.0287539
H	-50.2999515	23.8429497	-20.6580129
H	-49.9719966	24.6558949	-22.1806116
H	-52.5685506	26.9548044	-18.0657711
H	-47.7731796	28.2638140	-20.4360673
H	-59.6476097	35.1748561	-16.3199996
H	-55.7481002	34.2342042	-13.6851928
H	-53.2064146	37.8586169	-19.5158456

5FJC

O	-2.8220002	-5.6955499	4.1534669
C	-3.6290002	-4.5545499	3.9074669
C	-3.1000002	-3.7365499	2.7514669
O	-1.7180002	-3.3635499	2.9954669
C	-3.0640002	-4.4175499	1.3884669
O	-4.3260002	-4.4015499	0.7474669
C	-1.9990002	-3.6105499	0.6514669
O	-2.5230002	-2.3535499	0.2454669
C	-1.0050002	-3.3285499	1.7784669
N	0.0969998	-4.3165499	1.8494669
C	1.1499998	-4.2085499	0.9534669
O	1.1939998	-3.3605499	0.0824669
N	2.1449998	-5.1465499	1.0914669
C	2.2059998	-6.1525499	2.0314669
O	3.1729998	-6.9215499	2.0344669
C	1.0879998	-6.1935499	2.9224669
C	0.0989998	-5.2995499	2.8074669
N	1.5959998	4.0814501	-3.4675331
C	1.0419998	3.0254501	-4.3245331
C	-0.2820002	3.4204501	-4.9945331
O	-0.8160002	2.6704501	-5.8155331
O	-0.8470002	4.4884501	-4.7415331
C	0.8869998	1.6974501	-3.5805331
C	2.1819998	1.1194501	-3.0155331
S	1.9899998	-0.4555499	-2.1325331
C	1.1689998	-1.5055499	-3.3565331
C	0.6659998	-0.1375499	-0.9375331
C	1.2689998	0.5504501	0.2814669
O	1.7969998	1.7964501	-0.1215331
C	0.2659998	0.8934501	1.3714669
O	0.0899998	-0.1855499	2.2654669
C	0.8999998	2.1134501	2.0124669
O	1.6189998	1.7114501	3.1454669
C	1.8889998	2.6574501	0.9784669
N	1.5109998	4.0234501	0.5784669
C	2.2909998	5.1404501	0.6704669
N	1.5829998	6.1994501	0.2154669
C	0.3509998	5.7784501	-0.1585331
C	-0.7560002	6.4324501	-0.6785331
N	-0.7390002	7.7394501	-0.9015331
N	-1.8980002	5.7104501	-0.9815331
C	-1.9360002	4.3564501	-0.7575331
N	-0.8330002	3.7164501	-0.2275331

C	0.2949998	4.4094501	0.0544669
H	-3.6768899	-3.8908255	4.8036557
H	-4.6795418	-4.8246945	3.6383465
H	-3.7269804	-2.8184999	2.6659426
H	-2.6966597	-5.4593630	1.5417428
H	-1.5260265	-4.1628286	-0.1936635
H	-3.4542919	-2.5359383	-0.0101931
H	-0.5403117	-2.3301305	1.6391869
H	1.0733358	-6.9743860	3.6916477
H	-0.7801050	-5.3008141	3.4690172
H	2.5043343	4.4797593	-3.7498242
H	1.5879000	3.8863388	-2.4483041
H	1.7581621	2.8798080	-5.1616319
H	0.1541455	1.8663559	-2.7612255
H	0.4034030	1.0344056	-4.3260902
H	2.6590427	1.7636189	-2.2503834
H	2.9302819	0.8976645	-3.8062749
H	1.0281571	-2.4826910	-2.8539807
H	0.2003537	-1.0730625	-3.6673140
H	1.8549480	-1.6144534	-4.2174792
H	0.3084879	-1.1492114	-0.6391874
H	-0.1494776	0.4481883	-1.4017517
H	2.0585114	-0.1050611	0.7249109
H	-0.7065873	1.1733295	0.9171415
H	0.7781461	-0.0490001	2.9571913
H	0.1340084	2.8753410	2.2561453
H	1.2208987	2.0891176	3.9499850
H	2.9107873	2.6982786	1.4139707
H	3.3165472	5.1475548	1.0582824
H	0.0835814	8.2900532	-0.6582730
H	-2.8411536	3.7815199	-0.9983807
H	-3.2332144	-6.2177886	4.8643405
H	-4.3725516	-5.1590307	0.1368532
H	2.9244124	-5.0844247	0.4296645
H	-1.5696561	8.1927982	-1.2842037
H	0.7788192	4.8169992	-3.6508685

5FK1

O	2.3042010	-6.1950520	-3.4456852
C	3.1121978	-5.0310565	-3.3886853
C	2.5941998	-4.0630604	-2.3586895
O	1.2542050	-3.6280620	-2.7096881
C	2.4342005	-4.6110584	-0.9526950
O	3.6501956	-4.7030578	-0.2366978

C	1.4362044	-3.6410620	-0.3426975
O	2.0702019	-2.4160670	0.0013013
C	0.5072082	-3.3960630	-1.5306927
N	-0.6547874	-4.3140594	-1.5106927
C	-1.6497834	-4.0570605	-0.5796965
O	-1.6127836	-3.1200642	0.2053007
N	-2.7067792	-4.9320571	-0.5956964
C	-2.8657787	-6.0200528	-1.4286931
O	-3.8757746	-6.7200501	-1.3246936
C	-1.7937829	-6.2200519	-2.3586895
C	-0.7467871	-5.3850551	-2.3696895
N	-1.0357858	4.6939050	3.0682892
C	-0.6837872	3.4609100	3.7632867
C	0.7092073	3.5319098	4.3802840
O	1.1512056	2.5879133	5.0362814
O	1.4242045	4.5249059	4.2392846
C	-0.7647869	2.2669146	2.8232902
C	-1.8787826	1.2819186	3.1582888
S	-1.7207832	-0.2850754	2.2612923
C	-0.6797872	-1.2140716	3.4132880
C	-0.4857880	0.1109230	0.9952973
C	-1.1517854	0.5359215	-0.3066976
O	-1.8137829	1.7699167	-0.1056983
C	-0.1707894	0.7679204	-1.4436930
O	-0.0927896	-0.3570750	-2.2856895
C	-0.7597869	1.9339160	-2.2066901
O	-1.4267843	1.4529178	-3.3536854
C	-1.7697830	2.5729134	-1.2666937
N	-1.3437846	3.9429080	-0.9086951
C	-2.1277816	5.0659036	-0.9656950
N	-1.3937844	6.1288994	-0.5666965
C	-0.1527894	5.7079011	-0.2526978
C	0.9682062	6.3858986	0.2053007
N	0.9232064	7.7018933	0.4042996
N	2.1302017	5.6879013	0.4512994
C	2.1692016	4.3259065	0.2403003
N	1.0482059	3.6629090	-0.2136979
C	-0.1017896	4.3369064	-0.4596969
H	0.1060728	-5.5028484	-3.0423727
H	-3.4476414	-4.7565365	0.0758102
H	-1.8596703	-7.0660087	-3.0399204
H	0.1254313	-2.3658117	-1.5334316
H	0.8900558	-4.0627933	0.5187828
H	2.9487323	-2.6621141	0.3288485

H	1.9555456	-5.6025924	-1.0442074
H	3.2681571	-3.1878037	-2.3341552
H	3.1390973	-4.5115666	-4.3640402
H	4.1549777	-5.2695363	-3.1034023
H	-1.9849655	4.6731811	2.7090395
H	-0.4023962	4.8801448	2.2949031
H	-1.3527224	3.3418464	4.6319720
H	0.1921138	1.7312621	2.9133841
H	-0.8693229	2.6173912	1.7919752
H	-2.8732526	1.6727252	2.8951606
H	-1.8835132	1.0081041	4.2240316
H	-0.3732175	-2.1257459	2.8820862
H	0.1830675	-0.6030232	3.7141868
H	-1.3004782	-1.4759771	4.2802839
H	0.0320738	-0.8465199	0.8105124
H	0.2344601	0.8476224	1.3569106
H	-1.8663987	-0.2462893	-0.6226104
H	0.8261174	1.0320087	-1.0607941
H	-0.7065811	-0.1717650	-3.0171960
H	0.0243781	2.6528006	-2.4793111
H	-1.0929880	1.8944229	-4.1409074
H	-2.7567379	2.6489278	-1.7471178
H	-3.1671261	5.0711957	-1.2892356
H	0.0656224	8.2045562	0.2223238
H	3.0889416	3.7695200	0.4268537
H	3.6319125	-5.4967084	0.3056480
H	2.6366409	-6.7661460	-4.1446501
H	2.2536550	4.3636202	4.7194938
H	1.7446881	8.1862929	0.7387473

5FK2

O	-6.1888557	-2.4303437	-3.5040310
C	-5.0598610	-3.2783392	-3.3580320
C	-4.1238653	-2.7773420	-2.2860371
O	-3.6638677	-1.4433480	-2.6160354
C	-4.7198627	-2.6223424	-0.8980437
O	-4.7828622	-3.8423369	-0.1890471
C	-3.7948668	-1.6043474	-0.2510467
O	-2.5898728	-2.2253443	0.1729512
C	-3.4718683	-0.6953516	-1.4340409
N	-4.3278645	0.5166425	-1.4810408
C	-4.0968654	1.5156376	-0.5520454
O	-3.2408696	1.4356383	0.3089507
N	-4.9038613	2.6216325	-0.6500447

C	-5.9048568	2.8336316	-1.5750405
O	-6.5538538	3.8826265	-1.5440405
C	-6.0818560	1.7606367	-2.5070358
C	-5.3068595	0.6716418	-2.4330362
N	4.3160946	0.6056421	2.7109391
C	3.6300979	0.7926410	3.9859331
C	3.3870988	-0.5323525	4.7069297
O	2.5561030	-0.6303522	5.6119254
O	4.0260958	-1.5403476	4.4049310
C	2.3331038	1.5666376	3.7879340
C	1.2261092	0.7456415	3.1479371
S	0.0611149	1.7616367	2.2169416
C	-1.4708781	1.4786376	3.1319372
C	-0.1488844	0.6486420	0.8099483
C	0.5441123	1.2226392	-0.4130459
O	1.8291065	1.7026370	-0.0670475
C	0.7771115	0.1906441	-1.5030406
O	-0.3068833	0.1646442	-2.4010363
C	2.0381051	0.6836418	-2.1830374
O	1.6881067	1.3496387	-3.3740319
C	2.6491024	1.6916370	-1.2200422
N	4.0390958	1.3206387	-0.8810438
C	5.1120907	2.1726347	-0.9050437
N	6.2170853	1.4876376	-0.5370455
C	5.8710870	0.2086441	-0.2790465
C	6.6170833	-0.8923508	0.1289514
N	7.9290773	-0.7803512	0.3339506
N	5.9870863	-2.1043449	0.3209506
C	4.6300930	-2.2113443	0.1069516
N	3.9000964	-1.1133496	-0.2980463
C	4.5040932	0.0846444	-0.4910456
H	-5.4162248	-0.1819438	-3.1068993
H	-4.7462030	3.3579674	0.0309492
H	-6.8567935	1.8610060	-3.2642557
H	-2.4274175	-0.3515395	-1.3892094
H	-4.2684378	-1.0461963	0.5753942
H	-2.8510545	-3.1089627	0.4755052
H	-5.7239119	-2.1765743	-1.0183047
H	-3.2589725	-3.4627711	-2.2327877
H	-4.4890847	-3.3478529	-4.3023315
H	-5.3509688	-4.3053413	-3.0646584
H	4.9386566	1.3697209	2.4687654
H	3.6896368	0.4380635	1.9274460
H	4.2853936	1.3745564	4.6538269

H	2.5749759	2.4346502	3.1546928
H	1.9748751	1.9564481	4.7529456
H	0.6781194	0.1483584	3.8909753
H	1.6406676	0.0570005	2.3977010
H	-2.2879342	1.8583614	2.4998170
H	-1.5964030	0.4026258	3.3102099
H	-1.4001281	2.0377207	4.0742797
H	-1.2318599	0.5775081	0.6075449
H	0.2299843	-0.3414249	1.0949787
H	-0.0720328	2.0391038	-0.8343805
H	0.9439984	-0.8085620	-1.0710548
H	-0.0299503	0.7224876	-3.1480497
H	2.7286437	-0.1490354	-2.3719879
H	2.1616906	0.9693192	-4.1205724
H	2.6850620	2.6846739	-1.6946842
H	5.0528629	3.2234200	-1.1829538
H	8.3887788	0.1004640	0.1494983
H	4.1286605	-3.1693594	0.2533232
H	-5.5612893	-3.8368815	0.3758255
H	-6.7524587	-2.7904198	-4.1952561
H	4.6245462	-1.3323896	3.6541695
H	8.4653945	-1.5972553	0.5924703

5FK3

O	2.3105973	-6.1704060	-3.4629752
C	3.1385954	-5.0254086	-3.3229753
C	2.6045966	-4.0854107	-2.2739777
O	1.2675997	-3.6524116	-2.6299769
C	2.4375970	-4.6674094	-0.8839808
O	3.6485942	-4.7214092	-0.1619824
C	1.4025993	-3.7444115	-0.2609824
O	2.0035981	-2.5254142	0.1500168
C	0.5016014	-3.4554121	-1.4589795
N	-0.6913960	-4.3334102	-1.5079793
C	-1.6973938	-4.0984107	-0.5899816
O	-1.6403938	-3.2244126	0.2570167
N	-2.7873913	-4.9234088	-0.6819813
C	-2.9803907	-5.9454065	-1.5869792
O	-4.0233883	-6.6054051	-1.5439793
C	-1.8963931	-6.1254061	-2.5089771
C	-0.8193955	-5.3344079	-2.4409774
N	-1.0833951	4.1385708	3.1220103
C	-0.5003963	3.2185729	4.0980080
C	0.8876005	3.6355720	4.5780070

O	1.4345992	3.0365733	5.5030048
O	1.4955991	4.5735698	4.0690080
C	-0.4853963	1.7935761	3.5570092
C	-1.7933935	1.3965770	2.8890107
S	-1.7273937	-0.2484193	2.1390124
C	-0.7473959	-1.1734172	3.3430096
C	-0.4903963	0.0155801	0.8420152
C	-1.1433950	0.5755788	-0.4129818
O	-1.7223937	1.8345760	-0.1289825
C	-0.1763971	0.8065784	-1.5619793
O	-0.1723971	-0.2994192	-2.4309774
C	-0.7403959	2.0215756	-2.2609777
O	-1.4463943	1.6165765	-3.4119752
C	-1.7323937	2.6395742	-1.2879800
N	-1.3443945	4.0245711	-0.9519808
C	-2.1733927	5.1125687	-1.0109806
N	-1.4733942	6.2065662	-0.6359815
C	-0.2083971	5.8385670	-0.3409820
C	0.8976005	6.5625654	0.0880168
N	0.8146006	7.8795624	0.2810167
N	2.0885978	5.9055669	0.3150165
C	2.1735975	4.5415700	0.1170168
N	1.0676001	3.8335715	-0.3069822
C	-0.1093971	4.4685701	-0.5349816
H	0.0420437	-5.4430901	-3.1047394
H	-3.5306784	-4.7603547	-0.0098201
H	-1.9791489	-6.9170402	-3.2510068
H	0.1396577	-2.4166154	-1.4353559
H	0.8425533	-4.2120042	0.5669896
H	2.8835905	-2.7689400	0.4765538
H	1.9957948	-5.6735176	-1.0000843
H	3.2744875	-3.2082247	-2.2204258
H	3.2130567	-4.4683611	-4.2749289
H	4.1655366	-5.2953583	-3.0098086
H	-2.0939844	4.1321428	3.0806043
H	-0.6680735	4.0883432	2.1963226
H	-1.1184354	3.2582294	5.0093313
H	-0.2192206	1.1155722	4.3799740
H	0.3322309	1.7364577	2.8188223
H	-2.0530890	2.0537679	2.0482676
H	-2.6406523	1.3624032	3.5932281
H	-0.7335207	-2.2076995	2.9694541
H	-1.2657979	-1.1292219	4.3098258
H	0.2681621	-0.7677934	3.4069308

H	-0.0603941	-0.9777053	0.6299015
H	0.2910437	0.6877768	1.2217722
H	-1.9144357	-0.1358987	-0.7651529
H	0.8402511	1.0123660	-1.1938748
H	-0.7997697	-0.0686819	-3.1373790
H	0.0566048	2.7349324	-2.5088550
H	-1.0640193	2.0221000	-4.1967982
H	-2.7329071	2.6813488	-1.7446717
H	-3.2178933	5.0731921	-1.3146084
H	-0.0611958	8.3541524	0.1117744
H	3.1168853	4.0219423	0.2910445
H	1.6292992	8.3912518	0.5900299
H	2.3386716	4.6655767	4.5439898
H	3.6619783	-5.5217294	0.3707778
H	2.6755673	-6.7273081	-4.1570998

5FK4

O	-3.1866401	-5.5742436	3.7553994
C	-3.9116401	-4.3752436	3.5403994
C	-3.3446401	-3.6562436	2.3503994
O	-1.9346401	-3.4012436	2.5843994
C	-3.3606401	-4.4282436	1.0443994
O	-4.6246401	-4.3922436	0.3773994
C	-2.2036401	-3.7942436	0.2603994
O	-2.5746401	-2.5582436	-0.3206006
C	-1.2236401	-3.4642436	1.3763994
N	-0.1126401	-4.4282436	1.5153994
C	0.9563599	-4.3232436	0.6423994
O	0.9963599	-3.5082436	-0.2546006
N	1.9773599	-5.2072436	0.8403994
C	2.0343599	-6.1682436	1.8183994
O	3.0103599	-6.9092436	1.9083994
C	0.8903599	-6.2092436	2.6723994
C	-0.1166401	-5.3542436	2.5113994
N	1.8243599	4.1537564	-3.0646006
C	1.2403599	3.1427564	-3.9616006
C	-0.0346401	3.6677564	-4.6446006
O	-0.6416401	2.9497564	-5.4386006
C	0.9663599	1.8137564	-3.2476006
C	2.1853599	0.9077564	-3.2176006
S	2.0063599	-0.6102436	-2.2246006
C	0.7683599	-1.4852436	-3.2336006
C	0.8273599	-0.1892436	-0.9216006
C	1.5033599	0.4827564	0.2553994

O	2.1303599	1.7307564	-0.0546006
C	0.4873599	0.7607564	1.3453994
O	0.3943599	-0.3632436	2.1763994
C	1.0753599	1.9577564	2.0673994
O	1.6683599	1.5787564	3.2873994
C	2.1433599	2.5107564	1.1253994
N	1.8123599	3.9087564	0.8243994
C	2.6643599	4.9737564	0.9263994
N	1.9823599	6.0857564	0.5583994
C	0.7183599	5.7467564	0.2233994
C	-0.3616401	6.4897564	-0.2216006
N	-0.2466401	7.8067564	-0.3736006
N	-1.5626401	5.8557564	-0.4796006
C	-1.6606401	4.4957564	-0.3216006
N	-0.5736401	3.7597564	0.1033994
C	0.5933599	4.3727564	0.3973994
H	-1.0061593	-5.3474343	3.1464409
H	2.7630875	-5.1446191	0.2004831
H	0.8848934	-6.9548353	3.4654211
H	-0.7569903	-2.4877032	1.1734984
H	-1.7662430	-4.4791643	-0.4857991
H	-3.3166390	-2.7478246	-0.9098704
H	-3.1358956	-5.4868533	1.2464198
H	-3.8699519	-2.6896432	2.2198506
H	-3.8564238	-3.7079358	4.4201555
H	-4.9795637	-4.5700671	3.3246053
H	1.8427042	3.8629420	-2.0907229
H	2.7508846	4.4612620	-3.3397692
H	1.9138076	2.9631645	-4.8184733
H	0.1392404	1.3212701	-3.7818549
H	0.6254042	2.0589157	-2.2306997
H	3.0522388	1.4218940	-2.7727262
H	2.4758655	0.5445132	-4.2159522
H	0.5031794	-2.3851106	-2.6585128
H	-0.1016465	-0.8359476	-3.3857437
H	1.2287193	-1.7678669	-4.1893186
H	0.4698911	-1.1768922	-0.5783416
H	-0.0006206	0.3918176	-1.3463852
H	2.2531423	-0.2125723	0.6772396
H	-0.4946684	1.0222200	0.9219072
H	0.9462419	-0.1620324	2.9511598
H	0.2964379	2.7121812	2.2337292
H	1.2069753	1.9999897	4.0192456
H	3.1299552	2.4916864	1.6140999

H	3.6985942	4.9111021	1.2589216
H	0.6296498	8.2582692	-0.1511337
H	-2.6024295	3.9896719	-0.5390752
H	-5.0051593	-3.5160588	0.5128370
H	-3.6279590	-6.0866139	4.4394708
H	-1.0437275	8.3488567	-0.6782926
O	-0.5122920	4.9847634	-4.3578233
H	0.1357257	5.3242460	-3.7074654

5FK5

O	-2.7606078	-6.0636371	3.5034776
C	-3.5436058	-4.8916402	3.3154782
C	-2.9766069	-4.0076428	2.2294812
O	-1.6486108	-3.5606441	2.5964802
C	-2.7836078	-4.6666411	0.8744851
O	-3.9736044	-4.7086409	0.1154872
C	-1.6956108	-3.8176432	0.2344868
O	-2.2506089	-2.6256467	-0.2995118
C	-0.8396129	-3.4536442	1.4444832
N	0.3433834	-4.3336421	1.6074831
C	1.4323802	-4.1246424	0.7784852
O	1.4533802	-3.2696451	-0.0925124
N	2.5063772	-4.9556401	0.9914846
C	2.6043772	-5.9526372	1.9414821
O	3.6383742	-6.6226354	2.0204818
C	1.4413802	-6.1006371	2.7664796
C	0.3803832	-5.3066391	2.5794802
N	1.3853805	4.7313328	-3.1945037
C	1.2623812	3.5463359	-4.0425012
C	-0.2066148	3.3623365	-4.4494999
O	-0.9736128	4.3223339	-4.3855002
O	-0.6936138	2.3143394	-4.8824988
C	1.9513792	2.3323393	-3.4115028
C	1.1453812	1.0623429	-3.1685038
S	1.9803792	-0.0896538	-2.0575068
C	1.7883792	-1.6966492	-2.8575047
C	0.7113823	-0.1956535	-0.7785104
C	1.1863812	0.5733443	0.4394862
O	1.5923802	1.8703408	0.0614872
C	0.1353842	0.7883438	1.5094832
O	0.0923842	-0.2886531	2.4174806
C	0.6353827	2.0453402	2.1884812
O	1.3483807	1.6943411	3.3544782
C	1.6043802	2.6873383	1.2094842

N	1.1543812	4.0413346	0.8554852
C	1.8283792	5.2073313	1.1014842
N	1.0953812	6.2413284	0.6294856
C	-0.0356155	5.7513299	0.0874872
C	-1.1146124	6.3713279	-0.5265109
N	-1.1346124	7.6963243	-0.6585108
N	-2.1586096	5.5983302	-0.9955098
C	-2.1136098	4.2233339	-0.8495100
N	-1.0336128	3.6153359	-0.2365118
C	-0.0116156	4.3683338	0.2224869
H	-0.5345205	-5.3915965	3.1703968
H	3.3111818	-4.8202072	0.3873145
H	1.4522459	-6.8709187	3.5350369
H	-0.4614304	-2.4232640	1.3638952
H	-1.1099749	-4.3629175	-0.5249294
H	-2.9110411	-2.9111606	-0.9449971
H	-2.4384447	-5.7028917	1.0147676
H	-3.6327076	-3.1218687	2.1200077
H	-3.6027485	-4.2969221	4.2457262
H	-4.5769712	-5.1417061	3.0067222
H	1.2118985	4.5212822	-2.2133340
H	2.2828314	5.1977083	-3.2785741
H	1.7566710	3.7766093	-5.0008101
H	2.3281326	2.6634808	-2.4317049
H	2.8468559	2.0713340	-3.9979582
H	0.9086881	0.5355073	-4.1038128
H	0.1829016	1.2960217	-2.6980257
H	2.0275126	-2.4464077	-2.0879382
H	0.7527660	-1.8162909	-3.2027499
H	2.4962960	-1.7391676	-3.6957090
H	0.6012711	-1.2608226	-0.5146617
H	-0.2317739	0.1930741	-1.1830964
H	2.0302108	0.0226719	0.9010538
H	-0.8548858	0.9557272	1.0600044
H	0.7128051	-0.0456830	3.1249633
H	-0.1913327	2.7312524	2.4200673
H	0.9343113	2.0877208	4.1292145
H	2.6065915	2.7672273	1.6600087
H	2.7958547	5.2627478	1.5971721
H	-0.3792404	8.2442561	-0.2709008
H	-2.9364960	3.6138694	-1.2253700
H	-3.1934307	-6.6159961	4.1613947
H	-4.4899649	-3.9218324	0.3249822
H	-0.5021830	5.0832993	-3.9758740

H	-1.9420234	8.1479070	-1.0658877

5FK6

O	-6.2511261	2.3564699	3.6487792
C	-5.1461261	3.2314699	3.4817792
C	-4.2191261	2.7544699	2.3917792
O	-3.7281261	1.4264699	2.7087792
C	-4.8361261	2.5984699	1.0117792
O	-4.9321261	3.8234699	0.3077792
C	-3.9081261	1.5924699	0.3447792
O	-2.7251261	2.2294699	-0.1142208
C	-3.5441261	0.6854699	1.5207792
N	-4.3881261	-0.5295301	1.5777792
C	-4.1441261	-1.5275301	0.6497792
O	-3.2781261	-1.4405301	-0.2062208
N	-4.9481261	-2.6375301	0.7497792
C	-5.9531261	-2.8405301	1.6747792
O	-6.6001261	-3.8905301	1.6487792
C	-6.1401261	-1.7625301	2.6017792
C	-5.3721261	-0.6705301	2.5267792
N	5.0738739	-0.6115301	-3.1862208
C	3.8278739	-0.8085301	-3.9212208
C	3.4658739	0.4574699	-4.6952208
O	2.8118739	0.4104699	-5.7372208
O	3.8408739	1.5634699	-4.3042208
C	2.7208739	-1.2685301	-2.9742208
C	1.2818739	-1.0715301	-3.4402208
S	0.1388739	-1.9875301	-2.3772208
C	-1.4361261	-1.8225301	-3.2512208
C	-0.0661261	-0.7745301	-1.0532208
C	0.4868739	-1.2975301	0.2657792
O	1.7898739	-1.8215301	0.0887792
C	0.5998739	-0.2135301	1.3227792
O	-0.5071261	-0.2565301	2.1897792
C	1.8728739	-0.5505301	2.0717792
O	1.5598739	-1.0475301	3.3517792
C	2.5608739	-1.6355301	1.2587792
N	3.9398739	-1.2415301	0.9017792
C	5.0328739	-2.0665301	0.9317792
N	6.1178739	-1.3595301	0.5417792
C	5.7378739	-0.0935301	0.2567792
C	6.4408739	1.0274699	-0.1832208
N	7.7528739	0.9674699	-0.4052208
N	5.7738739	2.2174699	-0.3912208

C	4.4148739	2.2884699	-0.1662208
N	3.7278739	1.1724699	0.2657792
C	4.3698739	-0.0035301	0.4787792
H	-5.4882937	0.1863755	3.1940187
H	-4.7888297	-3.3789061	0.0752174
H	-6.9204719	-1.8656364	3.3530477
H	-2.4986430	0.3523796	1.4526153
H	-4.3944918	1.0250901	-0.4659831
H	-3.0168254	2.8923115	-0.7540952
H	-5.8529836	2.1873852	1.1070375
H	-3.3580305	3.4491080	2.3323196
H	-4.5658348	3.3231746	4.4189177
H	-5.4715539	4.2475597	3.1868733
H	5.8845435	-0.7690511	-3.7790620
H	5.1603559	-1.2134732	-2.3704665
H	3.9116959	-1.5671686	-4.7208162
H	2.8495520	-0.7331934	-2.0165076
H	2.8900029	-2.3329928	-2.7456856
H	1.1388918	-1.4692009	-4.4589658
H	0.9610257	-0.0195411	-3.4578193
H	-2.2181529	-2.0492620	-2.5092434
H	-1.4457067	-2.5548003	-4.0692427
H	-1.5463690	-0.7970091	-3.6291138
H	-1.1443517	-0.5745276	-0.9302058
H	0.4525790	0.1422682	-1.3684290
H	-0.1911086	-2.0774551	0.6583549
H	0.6857015	0.7841596	0.8622546
H	-0.1740968	-0.6455261	3.0174101
H	2.5098822	0.3413573	2.1346901
H	2.0053189	-0.5263320	4.0271645
H	2.6277493	-2.5647157	1.8464017
H	5.0044741	-3.1113759	1.2354879
H	8.2581761	0.1196009	-0.1887603
H	3.8817740	3.2277811	-0.3213083
H	-4.1622020	4.3568724	0.5371344
H	-6.9085863	2.7944388	4.1976041
H	4.4432503	1.4521519	-3.5361641
H	8.2489438	1.8081839	-0.6680500

5FKD

O	-2.4331782	-6.2257004	3.6065667
C	-3.2361782	-5.0587004	3.5125667
C	-2.7021782	-4.1037004	2.4755667
O	-1.3471782	-3.7177004	2.8135667

C	-2.5881782	-4.6507004	1.0605667
O	-3.8191782	-4.6167004	0.3615667
C	-1.5211782	-3.7587004	0.4405667
O	-2.0791782	-2.5167004	0.0365667
C	-0.5981782	-3.5047004	1.6355667
N	0.5898218	-4.3917004	1.6495667
C	1.5898218	-4.1617004	0.7185667
O	1.5318218	-3.2797004	-0.1224333
N	2.6708218	-5.0067004	0.7945667
C	2.8518218	-6.0377004	1.6965667
O	3.8778218	-6.7217004	1.6505667
C	1.7798218	-6.2077004	2.6315667
C	0.7168218	-5.3977004	2.5805667
N	0.1638218	4.9682996	-3.3524333
C	0.7178218	3.8342996	-4.0894333
C	-0.3371782	3.2432996	-5.0204333
O	-0.0181782	2.5822996	-6.0074333
O	-1.5351782	3.4352996	-4.8124333
C	1.2878218	2.7882996	-3.1304333
C	0.6598218	1.4002996	-3.1844333
S	1.7268218	0.1852996	-2.3734333
C	1.4288218	-1.3027004	-3.3604333
C	0.7238218	-0.1427004	-0.9074333
C	1.3818218	0.4612996	0.3215667
O	1.8188218	1.7772996	0.0395667
C	0.4198218	0.5692996	1.4885667
O	0.6288218	-0.4907004	2.3895667
C	0.7678218	1.8862996	2.1435667
O	1.4208218	1.6252996	3.3625667
C	1.7258218	2.5892996	1.1945667
N	1.2418218	3.9412996	0.8375667
C	2.0078218	5.0782996	0.8055667
N	1.2328218	6.1232996	0.4325667
C	-0.0231782	5.6712996	0.2245667
C	-1.1941782	6.3142996	-0.1654333
N	-1.1911782	7.6232996	-0.4064333
N	-2.3621782	5.5902996	-0.3034333
C	-2.3581782	4.2332996	-0.0534333
N	-1.1901782	3.6052996	0.3315667
C	-0.0361782	4.3042996	0.4725667
H	-0.1402014	-5.4948752	3.2516838
H	3.4119526	-4.8561593	0.1172844
H	1.8621696	-7.0047360	3.3675595
H	-0.2156161	-2.4728284	1.6293061

H	-0.9911417	-4.2527725	-0.3912582
H	-1.3683415	-2.0331666	-0.3914991
H	-2.1799252	-5.6755587	1.1352101
H	-3.3493622	-3.2088405	2.4549256
H	-3.2793294	-4.5259655	4.4806265
H	-4.2742410	-5.2959458	3.2106514
H	0.6491525	5.1524836	-2.4779132
H	0.1916054	5.8235099	-3.9029572
H	1.5272120	4.1234835	-4.7806947
H	1.2127660	3.1582684	-2.0961523
H	2.3643751	2.6809173	-3.3294505
H	0.5873825	1.0724991	-4.2358349
H	-0.3410591	1.3535452	-2.7362870
H	1.8337166	-2.1453146	-2.7798581
H	0.3520073	-1.4289156	-3.5365573
H	1.9738153	-1.1829754	-4.3063128
H	0.6919636	-1.2381549	-0.7683799
H	-0.2773417	0.2733179	-1.0850167
H	2.2315233	-0.1718758	0.6338265
H	-0.6278661	0.5809134	1.1445597
H	1.0688518	-0.0933794	3.1615439
H	-0.1390746	2.4880886	2.2909174
H	1.0027915	2.1132764	4.0794201
H	2.7088259	2.7135468	1.6752873
H	3.0681044	5.1061487	1.0507354
H	-0.3545613	8.1604626	-0.2244569
H	-3.2784781	3.6553989	-0.1512393
H	-2.0614463	8.0898464	-0.6239865
H	-1.6290675	4.0485397	-4.0479281
H	-2.8327947	-6.8182174	4.2500770
H	-3.7968788	-5.2907908	-0.3234189

5FKE

O	-2.3591154	-6.2346826	3.8782069
C	-3.3091128	-5.2136855	3.6372076
C	-2.8071142	-4.2636884	2.5892105
O	-1.4981179	-3.7526896	2.9372096
C	-2.6221147	-4.8626865	1.2122142
O	-3.8411113	-4.9496864	0.5112162
C	-1.6241174	-3.9026894	0.5732161
O	-2.2761157	-2.7136925	0.1552172
C	-0.7311200	-3.5626904	1.7672127
N	0.5078766	-4.3746882	1.8502126
C	1.5448736	-4.0466890	0.9922150

O	1.4788738	-3.1216914	0.1982171
N	2.6828705	-4.8116866	1.1242147
C	2.8728698	-5.8696837	1.9942121
O	3.9448668	-6.4806823	2.0002121
C	1.7468730	-6.1536832	2.8332097
C	0.6338761	-5.4186852	2.7372100
N	1.0378750	4.4852873	-3.3567729
C	0.5428765	3.4192903	-4.2217703
C	-0.8571197	3.7182894	-4.7567689
O	-1.3771182	2.9982914	-5.6087664
O	-1.5011179	4.6862866	-4.3507700
C	0.6088762	2.0652937	-3.5177723
C	2.0258722	1.5482955	-3.2747731
S	2.0648721	-0.0637004	-2.4447753
C	1.2228746	-1.0946974	-3.6707720
C	0.7858757	0.0922995	-1.1707790
C	1.3548740	0.6872976	0.1152173
O	1.8158728	2.0032941	-0.0917820
C	0.3478770	0.8042975	1.2502142
O	0.3168770	-0.3686994	2.0282120
C	0.8628756	1.9762943	2.0642120
O	1.5788736	1.5192955	3.1892087
C	1.7918729	2.7282920	1.1212147
N	1.2708744	4.0782884	0.8592152
C	1.9408726	5.2492848	1.0982147
N	1.1448747	6.2812820	0.7312157
C	-0.0241220	5.7812836	0.2702170
C	-1.1681188	6.3962816	-0.2217818
N	-1.2291185	7.7222780	-0.2957813
N	-2.2371158	5.6262837	-0.6327804
C	-2.1581160	4.2492876	-0.5537808
N	-1.0161192	3.6442896	-0.0637821
C	0.0378778	4.3962875	0.3432167
H	-0.2519343	-5.5946270	3.3544012
H	3.4559578	-4.5776187	0.5097908
H	1.8321691	-6.9747118	3.5421952
H	-0.4110332	-2.5121566	1.6929443
H	-1.0450133	-4.3552547	-0.2517935
H	-3.1608459	-2.9950137	-0.1252357
H	-2.1517266	-5.8558270	1.3321944
H	-3.5206338	-3.4233150	2.5095314
H	-3.5191578	-4.6433959	4.5612122
H	-4.2711348	-5.6130021	3.2625854
H	1.1781790	4.2190026	-2.3866514

H	1.8450389	4.9883108	-3.7059491
H	1.1538165	3.3676732	-5.1400328
H	0.0261073	1.3635885	-4.1341553
H	0.0847754	2.1929944	-2.5578609
H	2.6003510	2.2110436	-2.6101895
H	2.5980032	1.4007579	-4.2046396
H	1.1689124	-2.1048809	-3.2411757
H	0.2202593	-0.7046622	-3.8837037
H	1.8482159	-1.1045330	-4.5734519
H	0.4994097	-0.9499480	-0.9383865
H	-0.0788553	0.6410817	-1.5611694
H	2.1760013	0.0312679	0.4654237
H	-0.6547850	1.0301216	0.8570410
H	0.9598920	-0.2209056	2.7412281
H	0.0304645	2.6261035	2.3675952
H	1.1308252	1.7944554	3.9952933
H	2.7951044	2.8298909	1.5638340
H	2.9460527	5.3086116	1.5114333
H	-0.4521063	8.2750590	0.0389662
H	-3.0002825	3.6364864	-0.8785236
H	-2.7312627	-6.8628058	4.5042547
H	-3.7914254	-5.6787167	-0.1150997
H	-0.9557487	5.1895909	-3.7070423
H	-2.0757936	8.1682866	-0.6217775

5FKF

O	-6.3486627	2.4490744	3.3765834
C	-5.2386627	3.3140744	3.1925834
C	-4.2446627	2.7490744	2.2055834
O	-3.7996627	1.4300744	2.6115834
C	-4.7486627	2.5350744	0.7955834
O	-4.7576627	3.7270744	0.0525834
C	-3.7666627	1.5170744	0.2455834
O	-2.5446627	2.1510744	-0.1054166
C	-3.5186627	0.6460744	1.4715834
N	-4.3766627	-0.5659256	1.4915834
C	-4.0406627	-1.6389256	0.6665834
O	-3.0766627	-1.6379256	-0.0874166
N	-4.8726627	-2.7329256	0.7465834
C	-5.9826627	-2.8729256	1.5465834
O	-6.6356627	-3.9219256	1.5125834

C	-6.2596627	-1.7299256	2.3625834
C	-5.4746627	-0.6469256	2.3165834
N	4.5193373	-1.2069256	-3.2874166
C	3.3133373	-0.6539256	-3.8944166
C	3.4953373	0.7740744	-4.4224166
O	4.5963373	1.3380744	-4.4384166
O	2.5193373	1.3960744	-4.8544166
C	2.1553373	-0.7039256	-2.9054166
C	1.3363373	-1.9879256	-2.9514166
S	-0.2706627	-1.8139256	-2.1344166
C	-1.2216627	-0.9719256	-3.4214166
C	0.0033373	-0.4509256	-0.9584166
C	0.5313373	-0.9809256	0.3685834
O	1.7933373	-1.5519256	0.1005834
C	0.7793373	0.0380744	1.4835834
O	-0.3126627	0.1810744	2.3745834
C	1.9843373	-0.5289256	2.2105834
O	1.5503373	-1.2499256	3.3355834
C	2.5963373	-1.5419256	1.2575834
N	3.9873373	-1.2129256	0.9015834
C	5.0283373	-2.1099256	0.8785834
N	6.1523373	-1.4499256	0.5095834
C	5.8453373	-0.1529256	0.3015834
C	6.6193373	0.9230744	-0.0884166
N	7.9253373	0.7700744	-0.3244166
N	6.0283373	2.1610744	-0.2254166
C	4.6793373	2.3060744	0.0215834
N	3.9153373	1.2160744	0.4045834
C	4.4893373	0.0100744	0.5435834
H	-4.7024185	3.4973454	4.1543567
H	-5.5363421	4.3116090	2.7851938
H	-3.3724944	3.4435222	2.1661641
H	-5.7632509	2.0780823	0.8640286
H	-4.1805890	0.9222107	-0.6026001
H	-2.8080138	3.0377141	-0.4406291
H	-2.4627916	0.3022888	1.4991502
H	-7.1322282	-1.7769575	3.0245179
H	-5.6586757	0.2535874	2.9229609
H	4.8763565	-2.0962455	-3.6700138
H	4.5064393	-1.2506519	-2.2520364
H	3.0825968	-1.2600888	-4.7958715
H	2.5111876	-0.4918997	-1.8783352
H	1.5349615	0.1566127	-3.2452107
H	1.8239530	-2.8364806	-2.4298516

H	1.0994248	-2.3026169	-3.9882413
H	-2.1832053	-0.6887441	-2.9522425
H	-0.6745899	-0.0859005	-3.7995430
H	-1.4014176	-1.7055508	-4.2293720
H	-1.0144388	-0.0328203	-0.7913440
H	0.6416147	0.3272095	-1.4100915
H	-0.1876981	-1.7433251	0.7590217
H	1.0318675	1.0275917	1.0504834
H	-0.1866550	-0.5474602	3.0260263
H	2.7017380	0.2730497	2.4701428
H	1.7738883	-0.7616263	4.1488961
H	2.6164768	-2.5376956	1.7501314
H	4.9339100	-3.1698156	1.1424901
H	8.3679669	-0.1313854	-0.1495646
H	4.2038787	3.2902769	-0.0920136
H	8.4891253	1.5829527	-0.5748327
H	-4.6309327	-3.5256386	0.1444267
H	-6.9610274	2.8647382	4.0085806
H	-5.4496421	3.6718069	-0.6303150
H	5.2023639	-0.3817198	-3.5613737

5FKG

O	-2.7791688	-6.3303655	3.6471382
C	-3.7061688	-5.3003655	3.3651382
C	-3.1431688	-4.3753655	2.3251382
O	-1.8361688	-3.8813655	2.7081382
C	-2.9231688	-5.0053655	0.9691382
O	-4.1121688	-5.0803655	0.2211382
C	-1.8801688	-4.0793655	0.3461382
O	-2.4881688	-2.8903655	-0.1388618
C	-1.0231688	-3.7283655	1.5621382
N	0.1928312	-4.5683655	1.6941382
C	1.2678312	-4.2733655	0.8701382
O	1.2638312	-3.3373655	0.0891382
N	2.3768312	-5.0683655	1.0401382
C	2.5028312	-6.1363655	1.9031382
O	3.5568312	-6.7763655	1.9431382
C	1.3408312	-6.3943655	2.6981382
C	0.2528312	-5.6273655	2.5661382
N	1.5818312	4.3076345	-2.9418618
C	1.0918312	3.3606345	-3.9328618
C	-0.1721688	3.8636345	-4.6148618
O	-0.6381688	3.2676345	-5.5868618
O	-0.7421688	4.8816345	-4.2178618

C	0.8838312	2.0156345	-3.2488618
C	2.1958312	1.3636345	-2.8348618
S	1.9858312	-0.2343655	-2.0108618
C	1.1638312	-1.1713655	-3.3208618
C	0.5828312	0.0786345	-0.9068618
C	1.1208312	0.6516345	0.3991382
O	1.7108312	1.8976345	0.1061382
C	0.0928312	0.9416345	1.4771382
O	-0.0601688	-0.1443655	2.3601382
C	0.6948312	2.1316345	2.1991382
O	1.3518312	1.7136345	3.3731382
C	1.7158312	2.7256345	1.2431382
N	1.3298312	4.0976345	0.8761382
C	2.1008312	5.2106345	1.0581382
N	1.4178312	6.2826345	0.6051382
C	0.2188312	5.8796345	0.1451382
C	-0.8431688	6.5786345	-0.4108618
N	-0.7651688	7.8976345	-0.5638618
N	-1.9731688	5.8916345	-0.8008618
C	-2.0291688	4.5216345	-0.6308618
N	-0.9671688	3.8346345	-0.0758618
C	0.1478312	4.5026345	0.3041382
H	-0.6598994	-5.7820181	3.1501355
H	3.1782343	-4.8550466	0.4546302
H	1.3740844	-7.2266059	3.3983435
H	-0.6673024	-2.6868280	1.5057250
H	-1.2886952	-4.5814041	-0.4405702
H	-1.7763836	-2.3424434	-0.4804276
H	-2.4578022	-5.9975798	1.1226668
H	-3.8345873	-3.5236257	2.1962310
H	-3.9417855	-4.7180681	4.2752901
H	-4.6576202	-5.6874256	2.9535541
H	1.6576639	3.9527979	-1.9946001
H	2.4141192	4.8259236	-3.1982692
H	1.8099246	3.2364689	-4.7625703
H	0.3163166	1.3802877	-3.9455559
H	0.2529185	2.2148802	-2.3686394
H	2.7572385	1.9706334	-2.1118897
H	2.8514598	1.1488264	-3.6943977
H	0.9758787	-2.1729564	-2.9078238
H	0.2302705	-0.6809987	-3.6205812
H	1.8624123	-1.2453594	-4.1651552
H	0.1923968	-0.9312191	-0.6974338
H	-0.1732120	0.7078105	-1.3892193

H	1.8629273	-0.0550048	0.8201471
H	-0.8760476	1.2191776	1.0350503
H	0.5904703	0.0060881	3.0659770
H	-0.0829109	2.8727373	2.4289031
H	0.9018081	2.0685610	4.1466373
H	2.7075505	2.7725237	1.7189007
H	3.0969352	5.2037193	1.4970671
H	0.0527034	8.3889144	-0.2302924
H	-2.9237664	3.9768740	-0.9362981
H	-3.1722200	-6.9388337	4.2797006
H	-4.0003311	-5.7393581	-0.4697866
H	-1.5575216	8.4077151	-0.9305905
H	-0.2360558	5.2550283	-3.4624841

5FKH

O	6.3064101	-2.5284498	3.3865767
C	5.1194125	-3.3074483	3.3175769
C	4.1764143	-2.7914495	2.2605789
O	3.6814153	-1.4794519	2.6335782
C	4.7774131	-2.5774497	0.8805816
O	4.8724129	-3.7734474	0.1305831
C	3.8404149	-1.5484518	0.2635830
O	2.6544172	-2.1724505	-0.2044162
C	3.4844156	-0.6934535	1.4785806
N	4.3224142	0.5225442	1.5865802
C	4.0764144	1.5635421	0.7115820
O	3.2134163	1.5225421	-0.1504163
N	4.8794129	2.6625399	0.8765816
C	5.8814109	2.8205397	1.8135799
O	6.5234097	3.8715376	1.8405799
C	6.0704107	1.7005419	2.6865781
C	5.2994121	0.6195440	2.5465785
N	-4.3535689	0.5635441	-2.5704114
C	-3.8485699	1.0615432	-3.8434090
C	-3.6365704	-0.0684547	-4.8434071
O	-2.9735717	0.1105449	-5.8654051
O	-4.1175695	-1.1844525	-4.6484074
C	-2.5455725	1.8155414	-3.6324093
C	-1.4075746	0.9025434	-3.2064103
S	-0.1245773	1.7855417	-2.2944121
C	1.2984199	1.4825421	-3.3654100
C	0.1384223	0.6205440	-0.9344148
C	-0.4725766	1.1765428	0.3445827
O	-1.7835739	1.6395420	0.0865833

C	-0.5955763	0.1565449	1.4635806
O	0.4564216	0.3125446	2.3845788
C	-1.9105738	0.4915442	2.1335792
O	-1.6475742	1.0165432	3.4125767
C	-2.5625725	1.5545421	1.2605809
N	-3.9675697	1.2205428	0.9405816
C	-5.0115676	2.1095411	0.9725816
N	-6.1475654	1.4575422	0.6335823
C	-5.8505661	0.1645449	0.3765827
C	-6.6385644	-0.9114530	-0.0134166
N	-7.9505619	-0.7594533	-0.1934163
N	-6.0515655	-2.1434505	-0.2064162
C	-4.6945683	-2.2964503	-0.0134166
N	-3.9205697	-1.2224524	0.3705827
C	-4.4825687	-0.0044547	0.5645823
H	5.4122177	-0.2721604	3.1658944
H	4.7199705	3.4411688	0.2449576
H	6.8471599	1.7643931	3.4456537
H	2.4377002	-0.3604112	1.4353409
H	4.3152592	-0.9473577	-0.5314609
H	2.9378907	-3.0320420	-0.5520050
H	5.7719332	-2.1187121	1.0236667
H	3.3262986	-3.4927394	2.1815834
H	4.5791747	-3.3073832	4.2817954
H	5.3428556	-4.3596788	3.0560409
H	-4.3094890	1.2423489	-1.8203427
H	-3.9947749	-0.3347843	-2.2675458
H	-4.5821029	1.7446908	-4.3060459
H	-2.7305721	2.5696918	-2.8514352
H	-2.2626641	2.3576749	-4.5490522
H	-0.9453292	0.3849114	-4.0586162
H	-1.7973101	0.1648264	-2.4890545
H	2.1875307	1.8390422	-2.8247610
H	1.1402315	2.0655710	-4.2823209
H	1.3799328	0.4092121	-3.5807029
H	1.2284003	0.5211510	-0.7889287
H	-0.2891371	-0.3480022	-1.2280136
H	0.1630077	2.0012992	0.7166025
H	-0.6125182	-0.8719181	1.0658232
H	0.0455254	0.6677546	3.1921351
H	-2.5473807	-0.4022349	2.1819887
H	-2.1800914	0.5653985	4.0754190
H	-2.5774922	2.5159880	1.7995622
H	-4.9133567	3.1587571	1.2454225

H	-8.3708156	0.1486996	-0.0539294
H	-4.2305198	-3.2732500	-0.1586254
H	5.6974200	-3.7673482	-0.3631654
H	6.8366032	-2.8464583	4.1235251
H	-8.5110600	-1.5511585	-0.4766604
H	-3.8900288	-1.7426128	-5.4116305

6FZ0 (complete)

O	-1.54422270	4.34700590	-4.32115800
C	-2.67122270	5.19700590	-4.16015800
C	-3.14322270	5.26200590	-2.72415800
O	-3.53522270	3.94200590	-2.26115800
C	-2.12922270	5.72000590	-1.68815800
O	-1.93322270	7.12300590	-1.66215800
C	-2.71222270	5.16500590	-0.39415800
O	-3.74845230	6.01363140	0.10680400
C	-3.28222270	3.82700590	-0.87415800
N	-2.32322270	2.72400590	-0.64215800
O	-2.91822270	2.63800590	1.56584200
N	-1.32722270	1.21000590	0.81584200
C	-0.50722270	0.66100590	-0.14715800
O	0.26177730	-0.24899410	0.16684200
C	-0.65122270	1.23700590	-1.45115800
C	-1.53022270	2.22600590	-1.64915800
N	6.30577730	1.72500590	-1.08415800
C	5.01477730	1.31200590	-1.63415800
C	4.05177730	2.49500590	-1.61715800
O	2.98077730	2.45200590	-2.22215800
O	4.32377730	3.52800590	-1.00315800
C	4.46377730	0.13600590	-0.84115800
C	3.32677730	-0.65699410	-1.48315800
S	2.81477730	-1.99699410	-0.37215800
C	4.17977730	-3.16899410	-0.56815800
C	1.46977730	-2.88999410	-1.19615800
C	1.09377730	-4.11199410	-0.36815800
O	0.73777730	-3.69899410	0.95384200
C	-0.07822270	-4.93399410	-0.90315800
O	0.33277730	-6.24599410	-1.19015800
C	-1.12022270	-4.97499410	0.21484200
O	-1.59422270	-6.27199410	0.43384200
C	-0.37122270	-4.44999410	1.44584200
N	-1.20422270	-3.66099410	2.35884200
C	-2.07522270	-2.66099410	2.03784200
N	-2.64222270	-2.21299410	3.18084200

C	-2.14622270	-2.91299410	4.22284200
C	-2.38522270	-2.85699410	5.58984200
N	-3.25422270	-1.97899410	6.08084200
N	-1.71622270	-3.71699410	6.43484200
C	-0.81522270	-4.62499410	5.91784200
N	-0.58222270	-4.67499410	4.55784200
C	-1.23822270	-3.83199410	3.72084200
H	5.94842600	2.73743380	-0.73026840
H	5.15725860	1.01015850	-2.69275160
H	4.12835790	0.50582740	0.15373660
H	5.31198040	-0.56165190	-0.64588060
H	3.58531070	-1.08328710	-2.47403940
H	2.42550140	-0.01784970	-1.59768810
H	4.03664840	-3.96664300	0.18380220
H	4.17685590	-3.59099650	-1.59222130
H	5.13228160	-2.64656450	-0.36161300
H	1.95462620	-4.84224240	-0.36212990
H	-0.49102030	-4.44078670	-1.81469540
H	-0.32085080	-6.82695130	-0.73406480
H	-1.95160980	-4.28085830	-0.02700660
H	-2.51842770	-6.34688820	0.13564220
H	-0.01492010	-5.31527430	2.05407060
H	-2.21623920	-2.25404320	1.03105060
H	-3.73703190	-1.33936380	5.44995780
H	-3.42016130	-1.93612540	7.08654830
H	-0.27667670	-5.30929060	6.58856010
H	-1.23768250	4.41758490	-5.24160070
H	-1.05009510	7.29628610	-1.28944190
H	-0.01800330	0.86610820	-2.26440860
H	-1.67104740	2.71263780	-2.62817450
H	-4.20247570	3.57706630	-0.30726770
H	-1.17320060	5.19101720	-1.91659000
H	-2.44574260	6.25194390	-4.45109210
H	-3.53134300	4.85673090	-4.78558080
H	-1.93577430	4.99459740	0.38770070
H	-3.47571750	6.91748760	-0.16448470
H	-1.28897030	0.83325560	1.76856130
C	-2.21523750	2.20960820	0.61407640
H	6.66809310	1.17054870	-0.29103150
H	7.05232450	1.86406150	-1.78279840
H	-4.02497220	5.94423210	-2.68819570
H	1.79108488	-3.20213764	-2.16787187
H	0.61958476	-2.24721515	-1.29053947

6FZ0 (modified)

O	-1.54422270	4.34700590	-4.32115800
C	-2.67122270	5.19700590	-4.16015800
C	-3.14322270	5.26200590	-2.72415800
O	-3.53522270	3.94200590	-2.26115800
C	-2.12922270	5.72000590	-1.68815800
O	-1.93322270	7.12300590	-1.66215800
C	-2.71222270	5.16500590	-0.39415800
O	-3.74845230	6.01363140	0.10680400
C	-3.28222270	3.82700590	-0.87415800
N	-2.32322270	2.72400590	-0.64215800
O	-2.91822270	2.63800590	1.56584200
N	-1.32722270	1.21000590	0.81584200
C	-0.50722270	0.66100590	-0.14715800
O	0.26177730	-0.24899410	0.16684200
C	-0.65122270	1.23700590	-1.45115800
C	-1.53022270	2.22600590	-1.64915800
N	6.30577730	1.72500590	-1.08415800
C	5.01477730	1.31200590	-1.63415800
C	4.05177730	2.49500590	-1.61715800
O	2.98077730	2.45200590	-2.22215800
O	4.32377730	3.52800590	-1.00315800
C	4.46377730	0.13600590	-0.84115800
C	3.32677730	-0.65699410	-1.48315800
S	2.81477730	-1.99699410	-0.37215800
C	4.17977730	-3.16899410	-0.56815800
C	1.46977730	-2.88999410	-1.19615800
C	1.09377730	-4.11199410	-0.36815800
O	0.73777730	-3.69899410	0.95384200
C	-0.07822270	-4.93399410	-0.90315800
O	0.33277730	-6.24599410	-1.19015800
C	-1.12022270	-4.97499410	0.21484200
O	-1.59422270	-6.27199410	0.43384200
C	-0.37122270	-4.44999410	1.44584200
H	5.94842600	2.73743380	-0.73026840
H	5.15725860	1.01015850	-2.69275160
H	4.12835790	0.50582740	0.15373660
H	5.31198040	-0.56165190	-0.64588060
H	3.58531070	-1.08328710	-2.47403940
H	2.42550140	-0.01784970	-1.59768810
H	4.03664840	-3.96664300	0.18380220
H	4.17685590	-3.59099650	-1.59222130
H	5.13228160	-2.64656450	-0.36161300
H	1.95462620	-4.84224240	-0.36212990

H	-0.49102030	-4.44078670	-1.81469540
H	-0.32085080	-6.82695130	-0.73406480
H	-1.95160980	-4.28085830	-0.02700660
H	-2.51842770	-6.34688820	0.13564220
H	-0.01492010	-5.31527430	2.05407060
H	-1.23768250	4.41758490	-5.24160070
H	-1.05009510	7.29628610	-1.28944190
H	-0.01800330	0.86610820	-2.26440860
H	-1.67104740	2.71263780	-2.62817450
H	-4.20247570	3.57706630	-0.30726770
H	-1.17320060	5.19101720	-1.91659000
H	-2.44574260	6.25194390	-4.45109210
H	-3.53134300	4.85673090	-4.78558080
H	-1.93577430	4.99459740	0.38770070
H	-3.47571750	6.91748760	-0.16448470
H	-1.28897030	0.83325560	1.76856130
C	-2.21523750	2.20960820	0.61407640
H	6.66809310	1.17054870	-0.29103150
H	7.05232450	1.86406150	-1.78279840
H	-4.02497220	5.94423210	-2.68819570
H	1.79108488	-3.20213764	-2.16787187
H	0.61958476	-2.24721515	-1.29053947
H	-0.97909404	-3.87423121	2.11209234

6YLB (modified)

O	0.26088280	1.76714170	6.49675620
C	-0.87011720	1.09414170	6.00175620
C	-1.20911720	1.52714170	4.60075620
O	-1.55611720	2.94014170	4.57275620
C	-0.13611720	1.41214170	3.52575620
O	0.10088280	0.09214170	3.06575620
C	-0.72111720	2.30414170	2.43875620
O	-1.74911720	1.61714170	1.74075620
C	-1.31811720	3.44914170	3.27675620
H	-1.72334860	1.34453360	6.66795290
H	-0.75956840	-0.01896460	5.99713590
H	-2.08506480	0.91768960	4.25263460
H	0.81481370	1.84682350	3.90718670
H	0.07017720	2.65405400	1.73091360
H	-1.42512530	0.69437450	1.66040640
H	-2.26219060	3.80220810	2.79923680
P	1.60088280	-0.43885830	2.82075620
O	1.55488280	-1.88485830	2.54975620
O	2.46988280	0.09014170	3.88175620

O	2.08988280	0.24114170	1.46975620
C	1.29188280	0.17114170	0.29175620
C	1.78788280	1.08614170	-0.79824380
O	1.91188280	2.42814170	-0.25724380
C	3.15688280	0.72214170	-1.36024380
O	3.24488280	1.05014170	-2.74124380
C	4.08988280	1.61014170	-0.55624380
O	5.31688280	1.87814170	-1.19124380
C	3.24488280	2.87114170	-0.38624380
H	0.24165840	0.46426780	0.51933010
H	1.23802320	-0.87670630	-0.07967220
H	1.05238820	1.08171580	-1.63490500
H	3.38253750	-0.36104940	-1.19344160
H	4.22469890	1.14848240	0.44814920
H	5.95507670	1.19658470	-0.91534600
H	3.38073230	3.52364000	-1.28456370
C	3.06688280	-3.16485830	-0.82524380
C	1.71688280	-2.97985830	-1.50624380
S	0.32488280	-3.21885830	-0.37324380
C	0.63888280	-4.87985830	0.27775620
C	-1.02511720	-3.48585830	-1.55624380
C	-2.36211720	-2.94485830	-1.04524380
O	-2.24811720	-1.58785830	-0.59724380
C	-3.52311720	-3.01285830	-2.04124380
O	-4.46811720	-3.94285830	-1.57124380
C	-4.09211720	-1.60485830	-2.06624380
O	-5.32911720	-1.62085830	-1.39124380
C	-3.09011720	-0.71285830	-1.32924380
H	3.85668290	-2.78757500	-1.50313680
H	3.10648410	-2.59137650	0.12767940
H	1.57612550	-3.67346720	-2.36276160
H	1.57878410	-1.93946300	-1.86887900
H	1.36196730	-4.72266960	1.10237310
H	-0.31526660	-5.23877930	0.70798660
H	1.01437150	-5.56309920	-0.50703720
H	-0.72381180	-2.92678130	-2.46850290
H	-1.09973640	-4.56181830	-1.81406060
H	-2.67457460	-3.55994990	-0.17096530
H	-3.15516150	-3.30362030	-3.05715210
H	-5.31573700	-3.43749720	-1.53669530
H	-4.31120290	-1.23977350	-3.08746500
H	-5.16826260	-1.61858980	-0.42602230
H	-3.57434660	-0.00565390	-0.62200120
H	4.15819260	1.40302910	-2.84121120

H	3.52307420	3.45339180	0.51546600
H	-0.61197000	4.31074160	3.33243830
H	1.03816420	1.39349720	6.03038730
H	3.28117310	-4.22918610	-0.60814040
H	-2.48075160	-0.10784532	-1.96762680

6YLB (complete)

O	0.2608828	1.7671417	6.4967562
C	-0.8701172	1.0941417	6.0017562
C	-1.2091172	1.5271417	4.6007562
O	-1.5561172	2.9401417	4.5727562
C	-0.1361172	1.4121417	3.5257562
O	0.1008828	0.0921417	3.0657562
C	-0.7211172	2.3041417	2.4387562
O	-1.7491172	1.6171417	1.7407562
C	-1.3181172	3.4491417	3.2767562
H	-1.7233486	1.3445336	6.6679529
H	-0.7595684	-0.0189646	5.9971359
H	-2.0850648	0.9176896	4.2526346
H	0.8148137	1.8468235	3.9071867
H	0.0701772	2.6540540	1.7309136
H	-1.4251253	0.6943745	1.6604064
H	-2.2621906	3.8022081	2.7992368
P	1.6008828	-0.4388583	2.8207562
O	1.5548828	-1.8848583	2.5497562
O	2.4698828	0.0901417	3.8817562
O	2.0898828	0.2411417	1.4697562
C	1.2918828	0.1711417	0.2917562
C	1.7878828	1.0861417	-0.7982438
O	1.9118828	2.4281417	-0.2572438
C	3.1568828	0.7221417	-1.3602438
O	3.2448828	1.0501417	-2.7412438
C	4.0898828	1.6101417	-0.5562438
O	5.3168828	1.8781417	-1.1912438
C	3.2448828	2.8711417	-0.3862438
H	0.2416584	0.4642678	0.5193301
H	1.2380232	-0.8767063	-0.0796722
H	1.0523882	1.0817158	-1.6349050
H	3.3825375	-0.3610494	-1.1934416
H	4.2246989	1.1484824	0.4481492
H	5.9550767	1.1965847	-0.9153460
H	3.3807323	3.5236400	-1.2845637
C	3.0668828	-3.1648583	-0.8252438
C	1.7168828	-2.9798583	-1.5062438

S	0.3248828	-3.2188583	-0.3732438
C	0.6388828	-4.8798583	0.2777562
C	-1.0251172	-3.4858583	-1.5562438
C	-2.3621172	-2.9448583	-1.0452438
O	-2.2481172	-1.5878583	-0.5972438
C	-3.5231172	-3.0128583	-2.0412438
O	-4.4681172	-3.9428583	-1.5712438
C	-4.0921172	-1.6048583	-2.0662438
O	-5.3291172	-1.6208583	-1.3912438
C	-3.0901172	-0.7128583	-1.3292438
N	-2.2501172	0.1211417	-2.2092438
C	-1.8651172	-0.2038583	-3.4732438
N	-1.1181172	0.7961417	-3.9742438
C	-1.0131172	1.7571417	-3.0422438
C	-0.3571172	2.9701417	-3.0512438
N	0.3188828	3.3301417	-4.1392438
N	-0.4051172	3.7761417	-1.9382438
C	-1.1091172	3.3681417	-0.8282438
N	-1.7601172	2.1531417	-0.8332438
C	-1.7151172	1.3541417	-1.9222438
H	3.8566829	-2.7875750	-1.5031368
H	3.1064841	-2.5913765	0.1276794
H	1.5761255	-3.6734672	-2.3627616
H	1.5787841	-1.9394630	-1.8688790
H	1.3619673	-4.7226696	1.1023731
H	-0.3152666	-5.2387793	0.7079866
H	1.0143715	-5.5630992	-0.5070372
H	-0.7238118	-2.9267813	-2.4685029
H	-1.0997364	-4.5618183	-1.8140606
H	-2.6745746	-3.5599499	-0.1709653
H	-3.1551615	-3.3036203	-3.0571521
H	-5.3157370	-3.4374972	-1.5366953
H	-4.3112029	-1.2397735	-3.0874650
H	-5.1682626	-1.6185898	-0.4260223
H	-3.5743466	-0.0056539	-0.6220012
H	-2.1414189	-1.1292346	-3.9932354
H	0.4677776	2.6353935	-4.8697253
H	0.9195943	4.1527917	-4.0952823
H	4.1581926	1.4030291	-2.8412112
H	3.5230742	3.4533918	0.5154660
H	-0.6119700	4.3107416	3.3324383
H	1.0381642	1.3934972	6.0303873
H	3.2811731	-4.2291861	-0.6081404
H	-1.1516527	3.9987968	0.0694782

7JYY

O	-2.3800325	-5.9023392	-2.3020926
C	-2.4270325	-5.5733392	-0.9040926
C	-3.2630325	-4.3433392	-0.6490926
O	-2.5720325	-3.1613392	-1.1110926
C	-4.6150325	-4.2583392	-1.3380926
O	-5.5690325	-5.0453392	-0.6320926
C	-4.9230325	-2.7613392	-1.2640926
O	-5.6170325	-2.3413392	-0.1040926
C	-3.5230325	-2.1343392	-1.3220926
N	-3.2470325	-1.5043392	-2.6190926
C	-3.2510325	-0.1233392	-2.6720926
O	-3.4460325	0.5766608	-1.6920926
N	-3.0110325	0.4046608	-3.9160926
C	-2.7810325	-0.2873392	-5.0880926
O	-2.5800325	0.3366608	-6.1310926
C	-2.8020325	-1.7123392	-4.9510926
C	-3.0350325	-2.2593392	-3.7520926
N	0.2229675	-2.2623392	4.5259074
C	-1.0310325	-1.6753392	3.9859074
C	-2.2210325	-2.4863392	4.5089074
O	-2.1680325	-2.9383392	5.6619074
O	-3.2350325	-2.6923392	3.8409074
C	-1.0070325	-1.6533392	2.4509074
C	0.1529675	-0.8203392	1.9129074
S	0.0149675	-0.4193392	0.1519074
C	0.2409675	-2.0343392	-0.5760926
C	1.6609675	0.3006608	-0.0760926
C	1.7909675	1.6506608	0.6059074
O	3.1489675	2.1296608	0.4889074
C	0.9049675	2.7606608	0.0259074
O	-0.0510325	3.1916608	0.9869074
C	1.8959675	3.8706608	-0.3510926
O	1.4139675	5.1656608	-0.0680926
C	3.0779675	3.5296608	0.5439074
N	4.3379675	4.1336608	0.1249074
C	4.9669675	4.0176608	-1.0900926
N	6.0739675	4.7166608	-1.1720926
C	6.1659675	5.3676608	0.0509074
C	7.1069675	6.2756608	0.5809074
N	8.1739675	6.7146608	-0.0780926
N	6.8949675	6.7396608	1.8349074
C	5.8149675	6.3036608	2.4989074

N	4.8659675	5.4576608	2.1049074
C	5.0999675	5.0156608	0.8579074
H	-2.8913695	-6.3918828	-0.3074530
H	-1.4047024	-5.3991552	-0.4854875
H	-3.4344909	-4.2627524	0.4522619
H	-4.4992892	-4.5908837	-2.3921787
H	-5.4925863	-2.4357198	-2.1677956
H	-6.0102579	-3.1561638	0.2812787
H	-3.4587790	-1.3254742	-0.5655215
H	-2.6214640	-2.3208323	-5.8446024
H	-3.0053454	-3.3464902	-3.5779699
H	-0.2968172	-2.7053458	5.4274344
H	0.9834386	-1.6197710	4.7980386
H	-1.1116866	-0.6480021	4.3992207
H	-1.9967459	-1.2608946	2.1392051
H	-0.9807602	-2.6975429	2.0717657
H	0.1642908	0.1762237	2.4020478
H	1.1494719	-1.2931026	2.0528125
H	1.0399679	-2.5916099	-0.0510499
H	-0.7466243	-2.5559134	-0.5557397
H	0.5100312	-1.8807894	-1.6392666
H	2.4158127	-0.4220452	0.2981703
H	1.7990545	0.4058706	-1.1727056
H	1.5336475	1.5778987	1.6885349
H	0.3682067	2.3946827	-0.8867786
H	-0.0386771	4.1766058	0.9341277
H	2.2073971	3.7598712	-1.4119267
H	1.1486378	5.6081054	-0.8945690
H	2.8921293	3.9156616	1.5761325
H	4.5849808	3.3490162	-1.8741644
H	8.7979079	7.3766154	0.3827534
H	5.6980780	6.7148148	3.5188773
H	-2.1326636	-6.8402606	-2.3810171
H	-6.2969511	-5.2698863	-1.2406983
H	-3.0477724	1.4266535	-3.9840456
H	0.5894987	-3.0520635	3.9703302
H	8.3715205	6.3953333	-1.0246654
